# Inherited Anemia: A Contemporary Clinical and Lab Review

**DOI:** 10.3390/healthcare14203404

**Published:** 2026-10-12

**Authors:** Talal Qadah

**Affiliations:** 1 Department of Medical Laboratory Sciences, Faculty of Applied Medical Sciences, King Abdulaziz University, Jeddah 21589, Saudi Arabia; thqadah@kau.edu.sa; 2 Hematology Research Unit, King Fahd Medical Research Center, King Abdulaziz University, Jeddah 21589, Saudi Arabia; 3 Institute of Genomic Medicine Sciences, King Abdulaziz University, Jeddah 21589, Saudi Arabia

**Keywords:** inherited anemias, hereditary hemolytic anemia, laboratory diagnosis, molecular genetics, precision medicine

## Abstract

**Highlights:**

**What are the main findings?**

- The review indicates that the correct diagnosis of inherited anemias depends on an integrated pathway of clinical assessment, laboratory hematology, and molecular genetics, rather than a single diagnostic area. The clinical phenotype raises diagnostic suspicion, laboratory testing clarifies illness classification, and genetic sequencing provides molecular confirmation, especially in rare or phenotypically overlapping conditions.
- Modern laboratory and genomic techniques such as enhanced hematological testing, hemoglobin analysis, next generation sequencing, and long-read sequencing have greatly improved disease differentiation and molecular characterization. The data also show a change in treatment from standard supportive care to personalized, mechanism-based and precision-guided therapy.

**What are the implications of the main findings?**

- The paradigm allows for a patient-centered model of precision medicine, incorporating clinical observations and hematologic and genetic evidence. Such integration may increase diagnostic accuracy, separate overlapping phenotypes of inherited anemia, allow the interpretation of genotype-phenotype, and give a better basis for individualized therapeutic decision-making and long-term care of patients.
- The findings suggest progressive integration of molecular diagnosis into routine hematology, since genomic information increasingly impacts treatment decision-making. This combined approach can guide individualized transfusion and chelation regimens, disease-specific treatments, genetic counseling, and curative options such as hematopoietic stem cell transplantation and gene-based therapies.

**Abstract:**

Inherited anemias represent a diverse group of genetic disorders characterized by impaired red blood cell production, increased destruction, or abnormal hemoglobin synthesis. This review provides a comprehensive overview of the contemporary clinical landscape, diagnostic workflows, and therapeutic advancements for both major and rare inherited anemias. Major hemoglobinopathies, including thalassemia and sickle cell disease (SCD), continue to impose a significant global health burden, while rare conditions such as hereditary hemolytic anemias (HHAs), congenital dyserythropoietic anemias (CDAs), and Diamond–Blackfan anemia (DBA) present unique diagnostic challenges. Recent years have seen a paradigm shift in laboratory investigations, primarily driven by the integration of next-generation sequencing (NGS) and third-generation sequencing (TGS) into clinical practice. These technologies have revolutionized the identification of causative variants, particularly in complex or multiallelic cases that elude traditional biochemical assays. Furthermore, specialized flow cytometry, such as eosin-5-maleimide (EMA) binding, remains critical for the diagnosis of red cell membrane defects. Therapeutic management is also undergoing a transformation. Beyond traditional supportive care and hematopoietic stem cell transplantation (HSCT), groundbreaking gene-editing therapies, most notably CRISPR/Cas9-mediated BCL11A enhancer editing, offer the potential for curative outcomes in beta-hemoglobinopathies. Novel pharmacological agents, such as luspatercept for thalassemia and mitapivat for pyruvate kinase deficiency, are providing targeted options to improve erythropoiesis and reduce transfusion burden. Despite these advances, challenges remain in ensuring equitable access to these high-cost interventions and addressing the diagnostic gaps in resource-limited settings.

## 1. Introduction

Evaluation of inherited anemia and anemia in general is established to be based on clinical assessment, as therapy of the patient relies on the clinical manifestations and laboratory evidence rather than on the hematological levels. In another words, Anemia impacts clinical status when the oxygen-carrying capacity drops below an adequate threshold to meet oxygen consumption demand [1]. Clearly, inherited anemias are a heterogeneous group of genetically determined disorders characterized by deficiencies in hemoglobin synthesis, red blood cell (RBC) membrane stability, enzymatic activity, erythropoiesis, or iron metabolism that result in ineffective erythropoiesis, chronic hemolysis, or decreased oxygen transport. Hemoglobinopathies, thalassemia syndromes, enzymopathies, congenital dyserythropoietic anemias, hereditary membrane disorders, and other uncommon inherited hematological abnormalities can vary greatly in clinical manifestations and laboratory findings [2]. With major advances in hematology, the diagnosis of inherited anemias is still challenging because many other diseases have a similar clinical presentation and cryptic test results, often leading to delayed diagnosis or misdiagnosis.

According to the current research, a thorough diagnostic strategy that incorporates clinical evaluation, standardized laboratory hematology, targeted phenotypic investigations, molecular genetic analysis, and conventional hematological tests is necessary for an accurate diagnosis. Recent developments in next-generation sequencing (NGS), targeted gene panels, whole-exome sequencing, and genotype-phenotype correlation studies have considerably enhanced the precision of diagnoses, enabled precise disease categorization, facilitated tailored treatment strategies, offered genetic counseling, and facilitated family screening [3]. Hence, the modern care of hereditary anemia has progressively moved to a patient-centered approach of precision medicine that combines diverse clinical knowledge with advanced laboratory and genomic technology to improve patient outcomes [4].

Hereditary anemias have been extensively studied; however, the literature on the subject is divided between clinical medicine and laboratory hematology and molecular genetics. Many reviews deal with isolated topics, rather than giving an integrated perspective. This compartmentalized paradigm limits the capacity for clinicians and researchers to understand transdisciplinary diagnostic and treatment pathways necessary for precision medicine. In addition, the rapid development of molecular diagnostic technology and personalized therapy has created a gap between traditional clinical practice and current evidence. Therefore, a comprehensive narrative review that pulls together the available knowledge under a single conceptual umbrella is justified for accurate diagnosis, personalized management, and future research in inherited anemias.

This narrative review seeks to critically assess the recent advancements in hereditary anemias by integrating clinical medicine, laboratory hematology, and molecular genetics within a patient-centered precision medicine framework. In addition, it discusses normal erythropoiesis, disease-specific clinical presentations, laboratory diagnostic strategies, molecular genetic technologies, and novel therapeutic approaches, illustrating how these interrelated areas collectively facilitate accurate diagnosis, personalized treatment, enhanced clinical decision-making, and improved long-term patient outcomes in inherited hematologic disorders. The primary research question is as follows: “How do clinical medicine, laboratory hematology, and molecular genetics complement each other to provide accurate diagnosis, individualized treatment, and long-term care of hereditary anemias in the framework of precision medicine?”

The main contribution of this review is the presentation of a conceptual framework that integrates clinical medicine, laboratory hematology, and molecular genetics into a patient-centered precision medicine pathway, providing a holistic multidisciplinary perspective that advances the understanding, diagnosis, and treatment of inherited anemias beyond traditional evidence synthesis. Furthermore, this work also makes the following contributions:

- Provides a thorough multidisciplinary framework for linking diagnosis to management suited to the individual patient.
- Offers an overview of new medicines and developments in laboratory diagnostics and molecular genetics.
- Provides a roadmap for the translation of precision medicine in hereditary hematologic disease.

The remainder of the study is organized as follows: Section 2 shows the current clinical and diagnostic landscape of hereditary anemias. Section 3 shows the research materials and procedure. The analysis of the narrative review is presented in Section 4. The discussion is presented in Section 5. Section 6 presents the conclusion of the study.

## 2. The Current Clinical and Diagnostic Landscape of Inherited Anemias

There is a heterogeneous group of inherited anemias, caused by mutations in genes encoding proteins involved in the development, structure, and function of red blood cells (RBCs) [5]. These conditions are generally classified as hemoglobinopathies (mainly thalassemia and sickle cell disease), red cell membrane defects (hereditary spherocytosis), red cell enzymopathies (G6PD deficiency, pyruvate kinase deficiency), and disorders of erythropoiesis (congenital dyserythropoietic anemias, Diamond–Blackfan anemia) [6].

**Figure 1:**
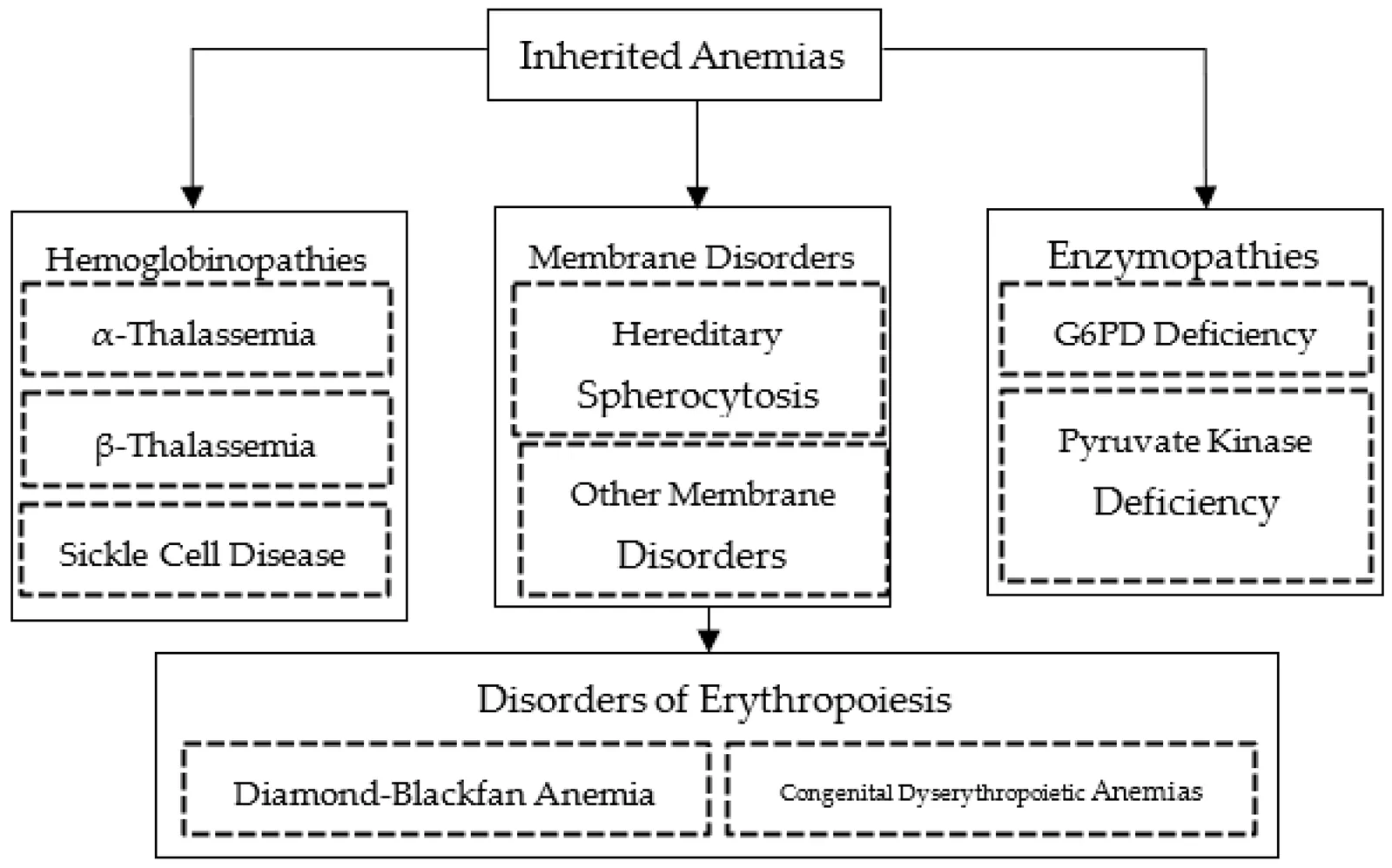
Classification of inherited anemias based on the affected red blood cell component.

Inherited anemias are a varied category of hereditary diseases affecting distinct structural and functional elements of red blood cells. While all these conditions lead to anemia, their molecular foundation, clinical presentations, and test features are quite different. Figure 1 offers an overview of the basic kinds of hereditary anemias according to their primary pathological abnormality and lays the basis for understanding their subsequent clinical presentation, diagnostic evaluation, and therapeutic therapy.

The clinical severity of these disorders ranges from asymptomatic carriers to life-threatening diseases requiring lifelong transfusion assistance. The diagnosis of red blood cell-related diseases and anemias, including hereditary hemolytic anemia, was traditionally based on clinical phenotype and biochemical testing, but trends in the development of diagnostic tools and the introduction of molecular diagnosis and molecular genetics have provided a better understanding of the underlying pathophysiology [7]. Personalized medicine, which uses genomic data to guide diagnosis and develop new targeted medicines, is becoming increasingly integrated into contemporary management [8].

The clinical appearance of thalassemia varies widely depending on the number of affected globin genes. Recent insights into thalassemia, from genomics to clinical practice, have helped explain this phenotypic variability. Thalassemia major is characterized by severe anemia that develops during infancy, as well as growth retardation, pallor, jaundice, and hepatosplenomegaly (Table 1).

Chronic erythroid marrow enlargement results in distinctive skeletal alterations and extramedullary hematopoiesis [9]. In contrast, thalassemia intermedia develops later in life, with moderate anemia that may not always necessitate regular transfusions, but patients are at risk for consequences such as osteoporosis and gallstones [9]. Thalassemia minor (trait) is usually asymptomatic but may present with mild anemia, fatigue, or exercise intolerance [9].

Sickle cell disease (SCD) is defined by a unique constellation of symptoms that are mainly caused by vaso-occlusion and chronic hemolysis [10]. Its clinical hallmarks include painful vaso-occlusive crises, jaundice, and an increased risk of stroke and splenic sequestration. Molecular testing in sickle cell disease, extending from newborn screening to transfusion care, is of utmost importance for early detection and the prevention of life-threatening consequences [10]. Moreover, recent developments in the diagnosis of sickle cell disease have improved the identification and clinical management of affected individuals [11].

Rare hereditary anemias share some but not all clinical characteristics. Genetics and genomics approaches for the diagnosis and research of hereditary anemias have improved understanding of their overlapping phenotypes [5]. Hereditary spherocytosis (HS), the most prevalent abnormality of the red cell membrane, classically presents with hemolysis, jaundice, gallstones, and splenomegaly [5,12]. Diamond–Blackfan anemia (DBA) is characterized by macrocytic anemia with morphological anomalies such as craniofacial or limb deformities [5].

Congenital dyserythropoietic anemias (CDAs) are characterized by ineffective erythropoiesis and bone marrow structural abnormalities, including erythroid hyperplasia. Recent updates on the clinical and laboratory aspects of hereditary dyserythropoietic anemias have further clarified their diagnostic characteristics [5,13]. Enzymopathies, such as pyruvate kinase [PK] deficiency and glucose-6-phosphate dehydrogenase (G6PD) deficiency, are characterized by non-spherocytic hemolytic anemia of variable severity, often worsened by oxidative stress or infections [12]. Their identification forms an important component of the diagnosis of congenital hemolytic anemias in adults [12].

An integrated approach is needed to differentiate among inherited anemias. The first clues are found in the basic laboratory data, including mean corpuscular volume (MCV) and mean corpuscular hemoglobin (MCH). For example, thalassemia carriers are frequently misdiagnosed as having iron-deficiency anemia because of their comparable microcytic indices. Therefore, the applications of next-generation sequencing in the screening and diagnosis of thalassemia are increasingly important for achieving diagnostic accuracy [14]. Nevertheless, clinical clues such the skeletal abnormalities of thalassemia or the limb deformities observed in DBA, are useful for establishing a differential diagnosis [5,9]. In cases with similar laboratory presentations, focused genetic testing and the impact of next-generation sequencing on the diagnosis and treatment of congenital anemias are important for differentiating disorders with overlapping characteristics [6].

**Figure 2:**
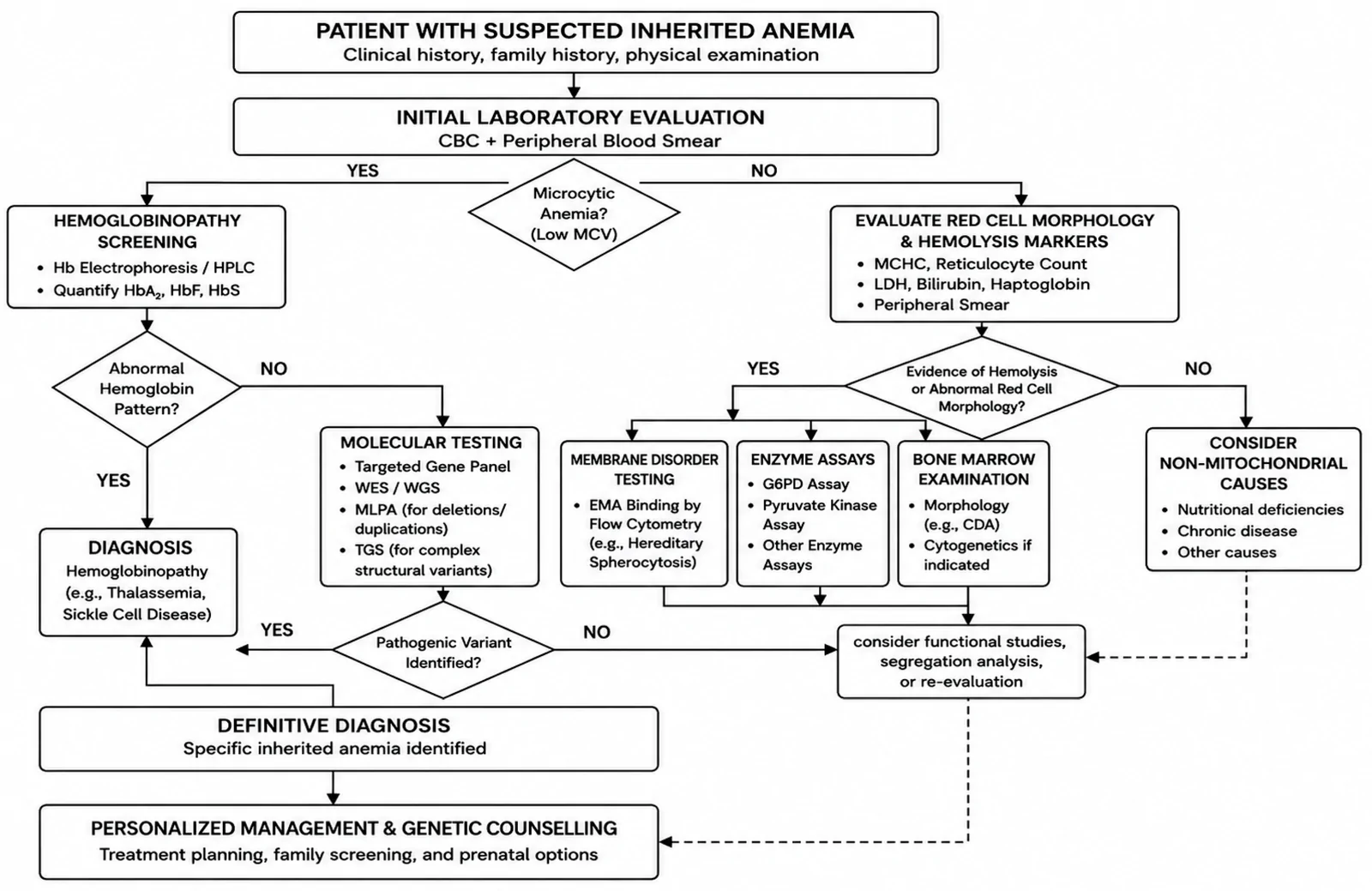
Diagnostic workflow for inherited anemias.

Inherited anemias represent a heterogeneous group of disorders with considerable clinical and laboratory overlap, making accurate diagnosis challenging but critical for optimal patient care. Modern diagnosis is therefore based on a structured stepwise approach that integrates clinical evaluation with hematological, biochemical, and genetic studies. The diagnostic workflow (Figure 2) progresses from routine laboratory evaluation to targeted testing and genomic analysis. This process incorporates molecular diagnosis of hereditary hemolytic anemias [2], next-generation sequencing in the diagnosis of rare inherited anemias [3], and broader genetics and genomics approaches for diagnosing hereditary anemias [5]. Ultimately, this integrated workflow facilitates accurate disease classification, confirmation of pathogenic variants, and personalized clinical management.

The routine investigations for diagnosis of inherited anemias include complete blood count (CBC), blood smear examination, and bone marrow studies [6]. In the case of hemglo-binopathies, high-performance liquid chromatography (HPLC) and isoelectric focus-ing (IEF) are the gold standards for quantifying hemoglobin variants (e.g., HbF, HbS, HbA2) [9,11]. Hemoglobin electrophoresis is a cornerstone test used in many diagnostic laboratories [15].

Membrane and enzyme defects require special tests to diagnose. Eosin-5-maleimide (EMA) binding by flow cytometry has become a favored method for the diagnosis of hereditary spherocytosis because of its high sensitivity and specificity compared to the traditional osmotic fragility test [12]. For enzymopathies, the deficiencies of pyruvate kinase or G6PD are confirmed through enzymatic activity assays [12]. Flow cytometry is also used to measure HbF containing RBC levels and to characterize red cell aggregates in SCD [11].

Next-generation sequencing (NGS) has revolutionized the diagnosis of hereditary anemias, especially uncommon anemias and complicated hemoglobinopathies [16,17]. NGS approaches such as targeted panels, whole-exome sequencing (WES), and whole-genome sequencing (WGS) allow simultaneous investigation of many genes [5,14]. This is especially useful in detecting causal variations when standard assays are unclear or confounded by recent transfusion [6].

Third-generation sequencing (TGS) is becoming a powerful tool for the detection of complex structural variations and large-scale deletions, especially in alpha-thalassemia, which are often difficult to characterize by standard NGS [17,18]. These molecular approaches have greatly improved the diagnostic yield and have led to the identification of novel causal genes in disorders such as CDA [8,13].

Prevention and early management require strong screening programs. This includes premarital screening, neonatal screening, and non-invasive prenatal diagnosis (NIPD) using cell-free fetal DNA from maternal plasma [14,19]. NIPD and preimplantation genetic testing (PGT) are used more and more to lower the birth rate of babies with severe hereditary anemias [18,19]. Building on these preventive approaches, Piel et al. [20] provide a comprehensive account of thalassemia, connecting disease mechanisms with diagnosis and management. Obeagu [21] highlights technologies advancing sickle cell anemia diagnosis, monitoring, and treatment, while Meenakumari et al. [22] emphasize molecular testing for thalassemia and appropriate diagnostic choices in resource-limited settings.

### Conceptual Framework of the Review

**Figure 3:**
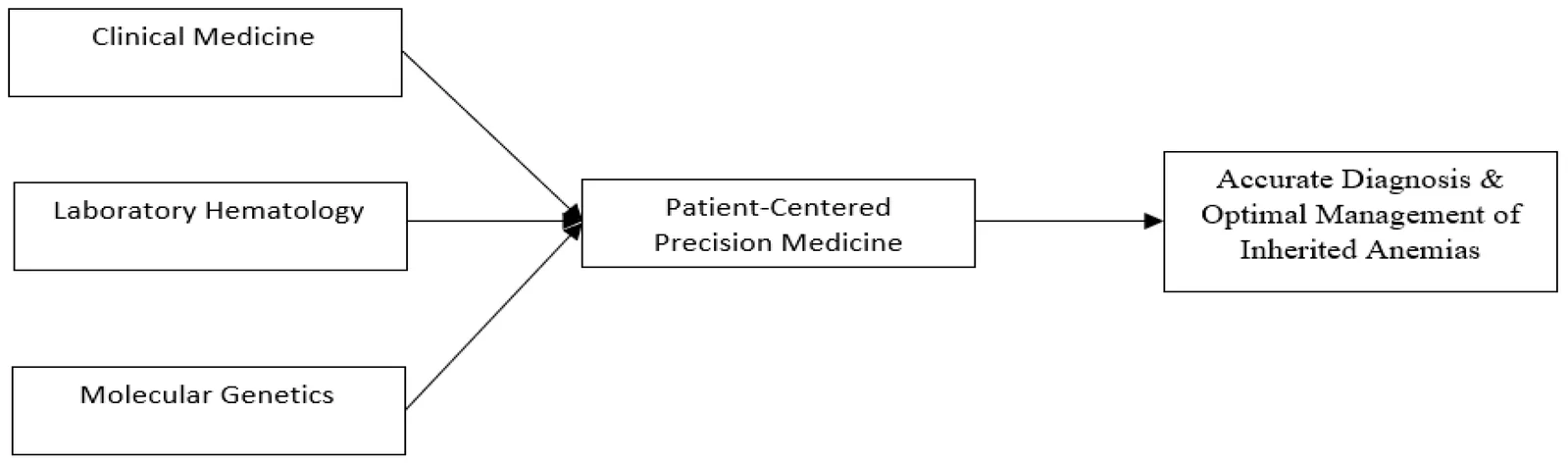
Conceptual framework for the integrated diagnosis and management of inherited anemias.

The present review adopts an integrative conceptual framework that links clinical medicine, laboratory hematology, and molecular genetics within a patient-centered precision-medicine pathway for inherited anemias (see Figure 3). The framework emphasizes that accurate diagnosis and individualized management cannot rely on any single disciplinary domain. Instead, clinical findings, laboratory phenotyping, and molecular confirmation provide complementary information that collectively supports diagnostic resolution, risk stratification, therapeutic selection, genetic counseling, and long-term patient management.

The framework begins with clinical medicine, laboratory hematology, and molecular genetics, each of which is an essential part of the patient evaluation process. Clinical medicine conducts the initial assessment by taking the patient’s history, conducting a physical examination, and recognizing characteristic clinical presentations. Laboratory hematology offers objective evidence in the form of routine and special hematological investigations such as complete blood count, blood smear analysis, hemoglobin electrophoresis, and biochemical assays. These findings are complemented with molecular genetics identifying disease-causing genetic variants through advanced genomic technologies such as targeted gene panels, next-generation sequencing (NGS), whole-exome sequencing (WES), whole-genome sequencing (WGS) and third-generation sequencing (TGS).

These three interrelated realms coalesce into the concept of patient-centered precision medicine, which is the basis of the framework. In precision medicine, clinical, laboratory, and molecular data are not investigated separately, but all available evidence about each patient’s disease is integrated to form a complete picture. This integrated method allows professionals to accurately classify the disease, differentiate between clinically overlapping hereditary anemias, provide customized therapies, provide genetic counseling, screen families, and predict the course of the disease. Thus, precision medicine centered on the patient is the bridge between the evidence for diagnosis and tailored clinical decision-making.

**Figure 4:**
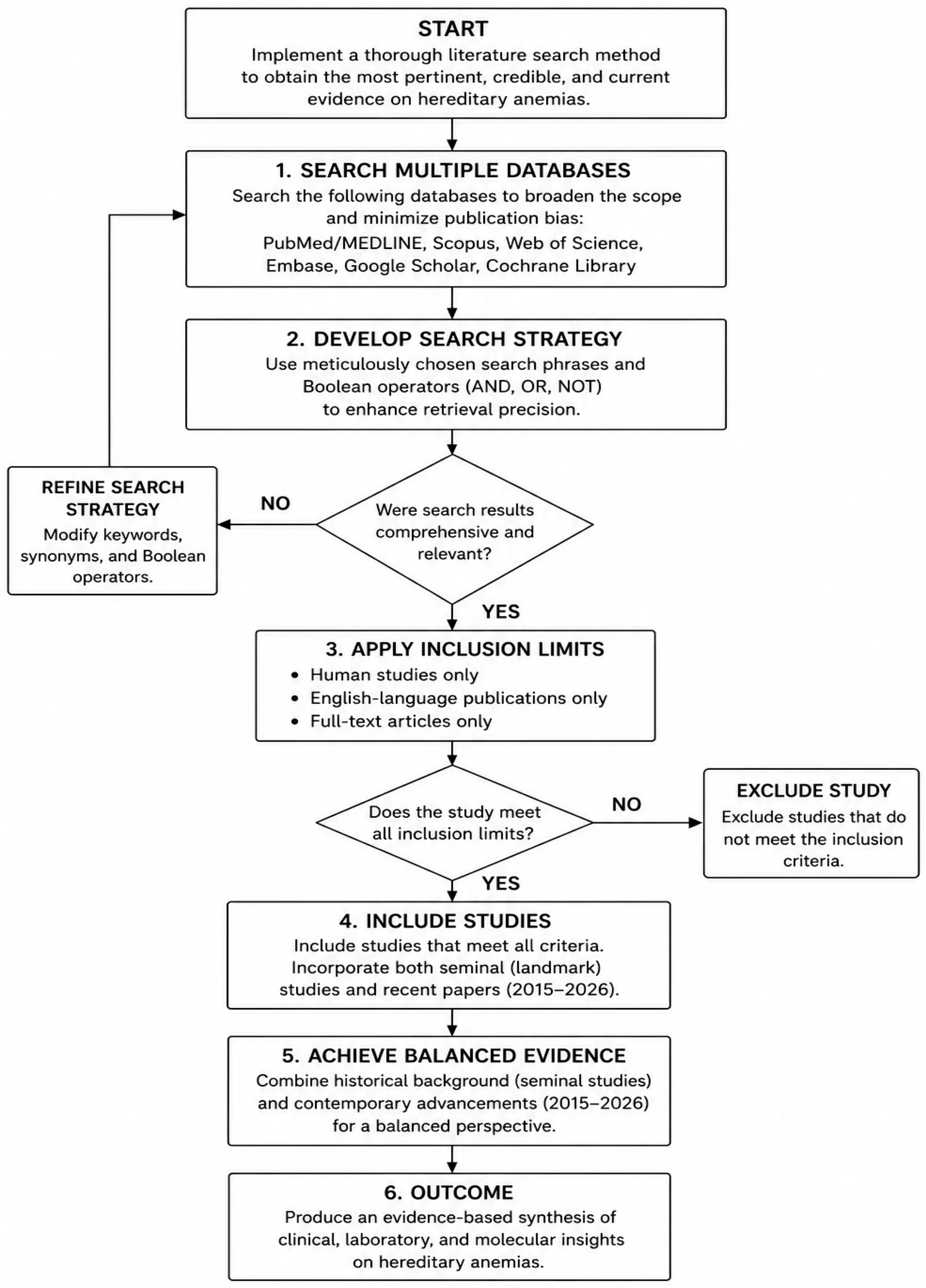
Study-selection flow diagram showing the identification, deduplication, screening, eligibility assessment, and final inclusion of publications in the narrative evidence synthesis.

To conclude, the framework shows that the successful management of inherited anemias is the result of coordinated multidisciplinary collaboration. Clinical expertise together with laboratory diagnosis and molecular genetics allows the clinician to shift from a symptom-based diagnosis to precise, mechanism-based treatment. This comprehensive approach enhances diagnostic accuracy and treatment effectiveness while also facilitating long-term patient monitoring, preventive strategies, and the wider application of precision medicine in hereditary hematological disorders, thus improving overall patient outcomes and quality of life. As a result, the methodology of implementing the framework is presented in Figure 4. It provides a transparent summary of the study-selection process used in this narrative review. It is a review-specific study-selection flow diagram and is not presented as an official PRISMA flow diagram.

## 3. Materials and Methods

The methodology flow is depicted in Figure 4. A thorough search of the literature was performed to include the most relevant, reliable, and up-to-date material on hereditary anemia. To minimize publication bias and to expand the spectrum of research articles considered, the author used many databases (PubMed/MEDLINE, Scopus, Web of Science, Embase, Google Scholar, and the Cochrane Library). The use of carefully selected search terms and Boolean operators helped to improve the accuracy of retrieval. The search was restricted to human studies, papers published in English, and full-text articles to ensure relevance and quality. Seminal research was included with current papers (2015–2026) to provide a historical background and contemporary developments, resulting in a balanced, evidence-based synthesis of clinical, laboratory, and molecular insights.

### 3.1. Information Sources and Search Strategy

This study was performed as a structured narrative review, integrating systematic literature-search and evidence-selection techniques to enhance the transparency and repeatability of evidence identification. This study was aimed at an integrative synthesis of disparate material on clinical, laboratory, molecular-genetic, diagnostic, and therapeutic aspects of hereditary anemias. The database search, duplicate removal, eligibility evaluation, research selection, and methodological appraisal were conducted in a methodical way, but the review was not designed as a formal systematic review or scoping review. Given the wide variety in inherited-anemia subtypes, study designs, diagnostic methodologies, therapies, demographics, and reported results, evidence was synthesized narratively.

A structured literature search was conducted in PubMed/MEDLINE, Scopus, Web of Science, Embase, Google Scholar, and the Cochrane Library. Reference lists of eligible articles, systematic reviews, guidelines, consensus documents, and landmark publications were also examined (see Table 2). The search combined Medical Subject Headings (MeSH), controlled vocabulary where available, and free-text terms covering general inherited-anemia terminology, hemoglobin disorders, hereditary hemolytic anemias, red-cell membrane disorders, red-cell enzyme disorders, congenital disorders of erythropoiesis, clinical features, laboratory diagnosis, and molecular genetics. Synonyms within concept groups were combined with OR and relevant concept groups with AND. The primary publication period was 2015–2026; earlier landmark studies were retained only when required for foundational physiological, diagnostic, or therapeutic context. The searches were limited principally to English-language human literature.

The search was organized around major concept groups reflecting the scope and conceptual framework of the review: (1) general inherited-anemia terminology; (2) hemoglobin disorders; (3) hereditary hemolytic anemias; (4) red-cell membrane disorders; (5) red-cell enzyme disorders; (6) inherited bone-marrow disorders; (7) clinical manifestations and complications; (8) laboratory diagnosis; and (9) molecular genetics and genomic diagnosis. Synonyms and spelling variants were combined using the Boolean operator OR within each concept group, while concept groups were combined using AND where appropriate. The principal publication period was 2015–2026, with older landmark publications retained when required to establish foundational physiological, diagnostic, or therapeutic concepts. The search was restricted principally to English-language human literature.

Search syntax was adapted to the indexing rules of each database. The literature search incorporated a broad range of terms to capture the major clinical, laboratory, and molecular dimensions of inherited anemias. General disease terms included “inherited anemia,” “inherited anaemia,” “hereditary anemia,” “hereditary anaemia,” “congenital anemia,” “congenital anaemia,” “genetic anemia,” and “genetic anaemia.” Hemoglobin-disorder terms included “hemoglobinopathy,” “haemoglobinopathy,” “thalassemia,” “thalassaemia,” “alpha-thalassemia,” “beta-thalassemia,” “alpha-thalassaemia,” “beta-thalassaemia,” “sickle cell disease,” “sickle cell anemia,” “HbS,” “HbC,” and “hemoglobin variants.” Hereditary hemolytic anemia terms included “hereditary hemolytic anemia,” “hereditary haemolytic anaemia,” “congenital hemolytic anemia,” “red blood cell disorders,” and “erythrocyte disorders.” Searches for specific disease categories incorporated membrane disorders (“hereditary spherocytosis,” “hereditary elliptocytosis,” “hereditary stomatocytosis,” and “red cell membrane disorders”), enzyme disorders (“G6PD deficiency,” “glucose-6-phosphate dehydrogenase deficiency,” “pyruvate kinase deficiency,” “red cell enzyme deficiency,” and “enzymopathy”), and bone marrow disorders (“congenital dyserythropoietic anemia,” “Diamond–Blackfan anemia,” “sideroblastic anemia,” and “iron metabolism disorders”). Clinical terms included “clinical presentation,” “clinical features,” “symptoms,” “diagnosis,” “management,” “complications,” and “patient outcomes.” Laboratory terms comprised “laboratory diagnosis,” “hematology,” “haematology,” “complete blood count,” “CBC,” “blood film,” “peripheral smear,” “hemoglobin electrophoresis,” “HPLC,” “enzyme assay,” “osmotic fragility,” “EMA test,” and “reticulocyte count.” Molecular-genetic searches included “molecular diagnosis,” “molecular genetics,” “genetic testing,” “gene panel,” “next-generation sequencing,” “NGS,” “whole exome sequencing,” “whole genome sequencing,” “genotype,” “phenotype,” and “precision medicine.”

The literature search was conducted iteratively from May 2026, with the final database search, updating, and evidence compilation completed on 31 July 2026. The re-porting and presentation of this structured narrative review were guided by the Scale for the Assessment of Narrative Review Articles (SANRA), particularly regarding the justification of the review, statement of aims, description of the literature search, referencing, scientific reasoning, and presentation of relevant evidence. Database-specific search strategies, including Boolean operators, field specifications, publication-period restrictions, document types, and language limits, are provided in Table 3.

### 3.2. Eligibility Criteria

Eligibility was assessed using predefined criteria (Table 4). Eligible evidence included human studies directly addressing inherited or hereditary anemias and providing substantive clinical, laboratory, molecular-genetic, diagnostic, or therapeutic information. Original studies, systematic reviews, meta-analyses, relevant narrative reviews, evidence-based guidelines, consensus statements, and selected landmark publications were considered. Acquired anemia without a hereditary basis, animal or in-vitro-only studies without direct clinical relevance, editorials, letters, incomplete conference abstracts, duplicate records, non-English publications, unavailable full texts, and studies without sufficient relevance to inherited anemia were excluded. Case reports were retained only when they documented a rare inherited disorder, novel variant, unusual phenotype, or clinically important observation not represented by larger studies.

### 3.3. Record Management, Screening, and Data Extraction

All records from the formal search and reference-list checking were consolidated into a single bibliographic dataset. Duplicate publications were identified by comparing DOI, title, authors, journal, and publication year, with manual verification where automated matching was uncertain. Screening proceeded sequentially through title, abstract, and full-text assessment. Two reviewers participated in the process: one performed the primary screening and extraction, and the second independently checked eligibility and extracted information for consistency; disagreements were resolved by consensus. A standardized extraction framework captured author/year, study design, population, inherited-anemia condition, clinical domain, laboratory or molecular method, intervention, principal findings, limitations, methodological-quality judgment, and role in the narrative synthesis.

It is important to distinguish the eligibility assessment from the subsequent methodological-quality appraisal. The reduction from 324 full-text publications to 69 publications resulted from application of the predefined eligibility, relevance, and evidence-integration criteria described above. Methodological appraisal was subsequently performed on the 69 retained publications as an additional quality-control stage and was used to determine the interpretive weight assigned to individual sources rather than to further exclude publications from the narrative synthesis.

#### Exploratory Search and Formal Selection Flow

The literature search consisted of an exploratory phase followed by a formal selection of studies. Searches were conducted iteratively from May 2026, and the final database search, update, and evidence compilation was finished on 31 July 2026. Broad database and search-engine queries provided exploratory searches of 279,226 hits and were used to assess the scope of the literature and refine the formal search approach. These hits shown were not unique bibliographic entries, since there was overlap among databases, search combinations, document versions, and Google Scholar results; hence, they did not represent the formal eligibility-screening population.

The official database searches resulted in 5486 records following the improvement of the search strategy. Reference-list checking discovered a further 214 records, resulting in 5700 records before de-duplication. A total of 4422 documents remained for title screening after removal of 1278 duplicates. Title screening resulted in 3498 exclusions and 924 records for abstract assessment. After abstract screening, 600 records were removed, and 324 publications were assessed for eligibility by full text. After full-text review, 255 publications were removed, leaving 69 papers for the final narrative synthesis.

Full-text exclusions were based on established eligibility and relevance criteria including insufficient inherited anemia-specific relevance, substantial thematic or evidential redundancy, insufficient substantive or methodological information for meaningful synthesis, and failure to contribute materially to the review’s predefined clinical, laboratory, molecular-genetic, diagnostic, or therapeutic objectives. Retrospective allocation of the 255 exclusions to fictitious reason-specific counts was not performed when the original screening record did not preserve mutually exclusive numerical counts for separate exclusion categories.

The final selection pathway was thus as follows: 5486 records from databases + 214 additional records = 5700 records; 5700 − 1278 duplicates = 4422 titles screened; 4422 − 3498 = 924 abstracts assessed; 924 − 600 = 324 full texts assessed; 324 − 255 = 69 publications included in the final narrative synthesis. Table 5 lists the database-specific search techniques and search parameters.

Studies that satisfied all stated inclusion criteria were included in the review (Table 6). Preferential publication of studies analyzing hereditary anemias from clinical, laboratory, molecular, or genetics points of view. Recent papers (2015–2026) were emphasized to capture recent advances in precision medicine, next generation sequencing, and enhanced laboratory diagnostics. However, seminal papers published before 2015 were included where they presented foundational concepts, historical background, or universally acknowledged principles of diagnosis that continue to inform current clinical practice.

Eligible articles were included to ensure the review synthesized high-quality material directly related to hereditary anemias. Following full-text assessment, 324 publications remained for detailed eligibility evaluation. Because the purpose of this narrative review was to develop an integrated synthesis rather than to include every publication addressing inherited anemia, an additional framework-based relevance assessment was applied. Publications were prioritized for final synthesis when they (1) directly addressed inherited or hereditary anemias; (2) provided substantive evidence relating to clinical medicine, laboratory hematology, molecular genetics, diagnosis, or therapeutic management; (3) contributed directly to at least one component of the proposed patient-centered precision medicine framework; (4) contained sufficiently detailed findings to support critical narrative synthesis; and (5) represented either contemporary evidence from 2015–2026 or landmark evidence essential for historical, physiological, diagnostic, or therapeutic context. Articles were excluded at this stage when their relevance to inherited anemia was peripheral, their findings substantially duplicated evidence already represented by stronger or more comprehensive publications, they lacked sufficient inherited-anemia-specific information, or they did not materially contribute to the review framework. After this final relevance assessment, 69 publications were retained for narrative evidence synthesis, while 255 full-text publications were excluded.

After research selection, the collected publications were synthesized using a narrative and thematic approach (Table 7). The evidence was presented in major themes of the review: classification of disease, clinical manifestations, laboratory diagnosis, molecular genetics, and management strategies. Rather than statistically aggregating the data, the research critically evaluated findings across individual research to discover consistent evidence, methodological variances, developing diagnostic technologies, and remaining gaps in knowledge. Thus, this approach enabled the integration of historical knowledge and modern advances, leading to a comprehensive understanding of inherited anemias.

Through database searching, reference-list checking, deduplication, and phased eligibility assessment, 5700 records were reduced to 69 publications retained for the final narrative evidence synthesis. All 69 publications satisfied the final eligibility and relevance criteria. Of these, 11 (15.9%) primarily addressed clinical medicine, 21 (30.4%) laboratory hematology, and 16 (23.2%) molecular genetics; the remaining 21 (30.4%) provided eligible cross-cutting or multidomain evidence and were not assigned exclusively to a single principal domain.

### 3.4. Methodological Quality Appraisal and Evidence Weighting

The 69 publications selected for final narrative synthesis were assessed for eligibility, and their methodological quality was further assessed. Given the varied nature of study designs in the evidence base, a critical-appraisal method sensitive to design was employed, rather than applying a single instrument to all publications. The AMSTAR 2 criteria were particularly applied to qualifying systematic reviews and meta-analyses covering protocol transparency, sufficiency of literature search, research selection procedures, risk-of-bias evaluation, and appropriateness of evidence synthesis. The appraisal of original observational studies was performed based on participant selection, sample size, validity of exposure and outcome assessment, confounding, statistical analysis, and completeness of reporting. When possible, randomized and nonrandomized experimental and interventional studies were evaluated for randomization, allocation techniques, comparability of groups, assessment of outcomes, missing data, and selective reporting.

The research examined clinical practice guidelines and consensus statements for transparency of production, interdisciplinary input, linking of advice to supporting data, and clarity of recommendations. The research evaluated narrative reviews for clarity of scope, evidence coverage, source selection transparency, scientific consistency, and relevance to hereditary anemia. Landmark and methodological papers were appraised for foundational contribution, ongoing relevance to science, and consistency with current evidence.

This was a quality-weighting stage, not an additional filter for eligibility; thus, all 69 papers remained in the final evidence set. The results of the appraisal were used to inform the relative interpretive weight attributed to specific publications and to highlight conclusions that should be treated with extra caution due to methodological constraints, indirectness, or inconsistency.

#### 3.4.1. Evidence Hierarchy and Weighting in the Narrative Synthesis

Due to the variety of the available literature, sources of evidence were not judged to have equal strength of evidence. Therefore, an evidence-weighting method was used during the narrative synthesis. The most weight was given to conclusions based on synthesis evidence (systematic reviews and meta-analyses), whereas recommendations on diagnosis and management were based on evidence-based clinical practice guidelines or well-developed consensus statements. The main empirical data came from high-quality original investigations of a clinical, laboratory, molecular, or diagnostic nature, especially when reporting diagnostic performance, genotype–phenotype connections, laboratory features, and therapy effects. Observational studies provided further real-world and disease-specific evidence but were evaluated with the understanding that they may be confounded and have design constraints.

Narrative reviews were utilized mainly for conceptual integration, historical background, and summaries of quickly developing topics and were cross checked, where possible, against original studies or higher-level evidence. Case reports and case series were deemed to be the lowest level of evidence and were included only when they described rare inherited disorders, novel genetic variants, unusual phenotypes, uncommon complications, or emerging therapeutic observations that could not be sufficiently captured by larger studies. The research relied on landmark publications prior to the primary review period to establish core principles, not to make present comparative effectiveness claims. Therefore, conclusions were drawn from the convergence of data from numerous sources, with more interpretive weight given to material that was methodologically stronger and more directly relevant.

Methodological quality and strength of evidence guided the interpretation of evidence supporting the suggested framework. In the conclusions related to clinical medicine, priority was given to guidelines, systematic reviews, and important clinical studies; conclusions for laboratory hematology were concentrated on diagnostic and laboratory investigations with proven analytical or clinical validity; and conclusions in molecular genetics were biased toward modern genomic studies, good practice recommendations, and systematic evidence. Isolated reports and narrative assessments provided useful context but were not permitted to override stronger evidence. Therefore, the approach captures convergence across diverse streams of evidence rather than an unweighted aggregation of varied articles.

#### 3.4.2. Assessment of Evidence Consistency and Confidence

This research devised a qualitative interpretive-confidence framework to describe the level of confidence in the main findings of the narrative synthesis. It was not designed to replace GRADE or any other validated evidence-certainty system. Each judgment was informed by four considerations: the methodological quality of the major supporting evidence, consistency of findings across relevant sources, directness to the review purpose and conclusion, and overall evidence strength and sufficiency.

These domains were not given any numerical score or weight. We judged that there was high interpretive confidence when the major evidence was methodologically solid, widely consistent, directly applicable and sufficiently developed (see Table 8). We assessed moderate interpretive confidence when a conclusion was widely supported but one or more categories had important limitations (e.g., methodological problems, some inconsistency, indirectness, or limited evidence). We had limited interpretive confidence in cases where multiple domains were substantially limited or where evidence was sparse, inconsistent, indirect, or methodologically weak. These categories are qualitative assessments relevant to the review and are not GRADE ratings or a standardized system for the classification of certainty of evidence.

These confidence judgments directly affected the strength of the terminology utilized in the synthesis. High-confidence conclusions are presented as established or strongly supported findings; moderate-confidence conclusions are interpreted cautiously and recognize heterogeneity or methodological limitations; and limited-confidence findings are described as preliminary or emerging and are not used independently to support definitive clinical recommendations. Accordingly, information related to proven laboratory and molecular diagnostic procedures was given more interpretive weight than evidence related to developing therapeutic modalities for which comparative effectiveness, long-term safety, or durability has not yet been fully demonstrated.

## 4. Narrative Review Synthesis

**Figure 5:**
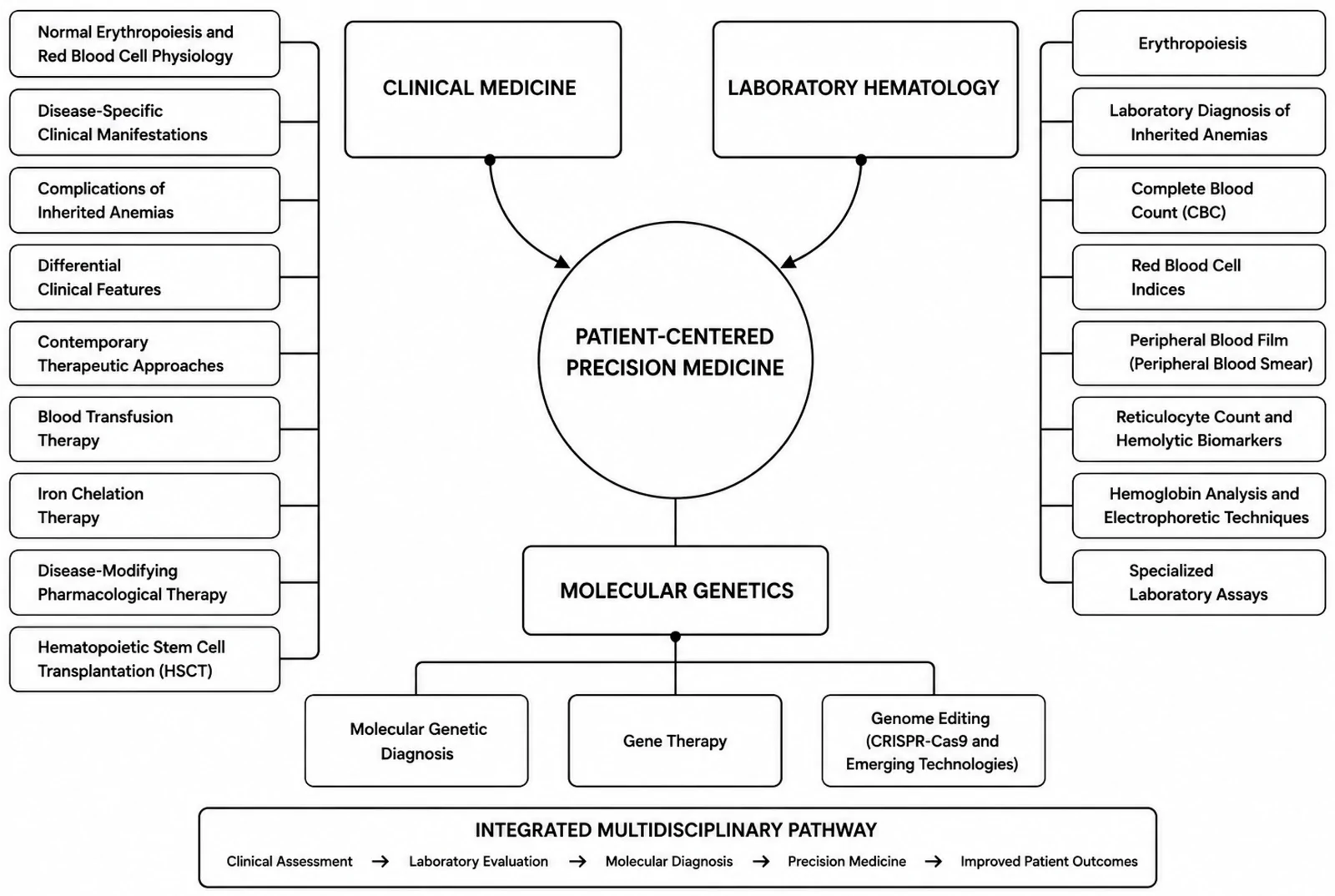
The narrative evidence synthesis flow.

A narrative evidence synthesis was chosen as the design, as objectives, patient demographics, diagnostic procedures, and reported outcomes of the included research varied greatly. Figure 5 depicts the conceptual framework of this narrative review classifying the several areas relevant to diagnosis and treatment of inherited anemias. The framework presents the combined input of clinical medicine, laboratory hematology, and molecular genetics for patient-centered precision medicine. This conceptual framework serves to guide a systematic review of the individual sub-topics within these domains, a critical appraisal of the existing evidence, and an illustration of how the integration of these factors improves diagnostic accuracy, personalized treatment approaches, and long-term patient outcomes in inherited hematological disorders.

Of the 69 publications constituting the final evidence base, 48 (69.6%) mapped directly to one of the three principal framework domains: clinical medicine (n = 11), laboratory hematology (n = 21), and molecular genetics (n = 16). The remaining 21 publications (30.4%) provided eligible cross-cutting evidence relevant to more than one framework component, including disease classification, pathophysiology, integrated diagnosis, and therapeutic management. These 21 publications remained part of the evidence base because they satisfied the predefined eligibility and relevance criteria. In contrast, references cited solely for general background, historical context, methodological explanation, or regulatory information were not treated as included evidence publications and were excluded from the n = 69 evidence count.

### 4.1. Clinical Perspectives of Inherited Anemias

Clinical assessment establishes the pre-test probability of an inherited anemia, defines disease severity, and identifies complications that influence subsequent laboratory and molecular testing. Its principal limitation is lack of specificity: persistent anemia, jaundice, splenomegaly, growth impairment, and transfusion dependence occur across several inherited disorders. Consequently, clinical findings are most informative when used to guide rather than replace phenotypic laboratory testing and molecular confirmation.

#### 4.1.1. Erythropoiesis and Red Blood Cell Physiology

Normal erythropoiesis and red blood cell physiology is the regulated production, maturation and function of red blood cells. Erythrocytes are derived from hemat-poietic stem cells and over their lifespan they keep appropriate oxygen transport, tissue oxygenation and normal hematologic homeostasis. Many past re-search projects have been performed within the subject of Erythropoiesis. A key work in this regard is that of Lee et al. [23] who studied the different functions of HIF1α and HIF2α in metabolism and erythropoiesis regulation through three genetically modified mice models. HIF1α decreased mitochondrial function and exercise capacity, while HIF2α preserved metabolism and boosted erythropoietin production. Polycythemia was abolished by muscle-specific deletion of EPO, demonstrating that skeletal muscle regulates systemic erythropoiesis through the PHD-HIF2α-EPO pathway.

The molecular causes of inefficient and faulty erythropoiesis were summarized by El Hoss et al. [24]. Apoptosis, inflammation, oxidative stress, globin imbalance and clonal mutations were identified in the review as major contributors to erythroid failure. Emerging possibilities to address defective erythropoiesis in hereditary anemias include pharmaceutical strategies such as luspatercept, anti-inflammatory treatments, metabolic modulators and gene editing methods.

Nguyen et al. [25] studied 1485 pregnant women to explore associations of vita-min D, body mass index, eating patterns and mi-cronutrients associated with erythropoiesis. Vitamin D was positively associated with iron, folate and vitamin B12, and these interactions were largely mediated by BMI and dietary habits. The results suggest that, besides genetic and molecular mechanisms, dietary and maternal variables in-fluence erythropoietic capacity.

Olver [26] reviewed the process of erythropoiesis, from stem-cell differentiation to mature erythrocyte formation, using data obtained from humans and animal models. Erythroid maturation is coordinated by growth-factor signaling, kinase activation, transcription factors and erythroblastic islands in the bone marrow microenvironment. This chapter provides an important cellular framework for understanding how inherited mutations perturb specific stages of erythroid development.

Caulier and Sankaran [27] studied the molecular and cellular regulation of human erythropoiesis. They stressed the combined roles of cytokine signaling, transcriptional and post-transcriptional control, and human genetics in sustaining normal red cell output. The review also identified BCL11A as a critical regulator of hemoglobin switching and highlighted single-cell technologies as prospective approaches to im-prove diagnosis and develop targeted therapeutics.

Dzierzak and Philipsen [28] review illustrating differences between embryonic and adult erythropoiesis. Production of ~2 × 10^11^ erythrocytes per day arises from adult progenitors. This provides the developmental framework for explaining hereditary erythroid disorders. Similarly, Sridharan and Sivaramakrishnan [29] carried up a Network meta-analysis of 136 studies (102,882 participants). SGLT2 inhibitors in-creased hemoglobin, hematocrit, RBC count and EPO but did not decrease anemia; certainty of evidence was low. Further examination by Na-Phatthalung et al. [30] pro-pose Mouse knockout tests demonstrated that osteoblast-derived ERFE regulated stress erythropoiesis via BMP4 signaling, EPO responsiveness and erythroid growth. Eryth-ropoietin-stimulating agents in heart failure with anemia: a complete review. Bunting et al. [31] The passage describes methods but does not offer pooled clinical outcomes. Finally, a randomized experiment by Salgado et al. [32] indicated that recombinant EPO enhanced hemoglobin bulk, but decreased endogenous erythropoietic adaptability following high-altitude exposure.

Munkongdee et al. [33] described conventional laboratory methods including hemato-logical indices, hemoglobin analysis, electrophoresis, HPLC, and molecular tests. Meenakumari et al. [22] pointed out the necessity of low-cost molecular diagnostic approaches for resource-limited settings, and Tang et al. [18] showed the advantage of third-generation sequencing for detection of complex anomalies in globin genes. Soliman et al. [34] pointed out the need of genetic diagnosis for the disease monitoring and long-term follow-up. Besides hemoglobinopathies, the classification, molecular mechanisms and laboratory features of congenital dyserythropoietic anemias have been updated by Russo et al. [13] and hereditary hemolytic anemias, including membrane, enzyme and hemoglobin disorders. Evidence-based transfusion protocols and iron chelation techniques to decrease the consequences of transfusion were also outlined by Taher et al. [35]. Cappellini et al. [36] proposed internationally accepted guidelines for the management of transfusion-dependent thalassemia, with standardized recommendations for clinical practice.

The distinction between defective and ineffective erythropoiesis is particularly important for interpretation of reticulocyte responses and emerging therapies. El Hoss et al. [24] identified apoptosis, oxidative stress, inflammation, globin-chain imbalance, and dysregulated signaling as recurrent mechanisms of erythroid failure. However, mechanistic findings from non-inherited conditions and experimental models should not be extrapolated directly to inherited anemia without disease-specific validation. For that reason, the present synthesis gives greater weight to human inherited-anemia studies and uses general erythropoiesis literature only as contextual evidence.

#### 4.1.2. Disease-Specific Clinical Manifestations

Disease-specific clinical manifestations are signs, symptoms, and physical findings that are uniquely associated with each inherited anemia and allow for clinical recognition, differential diagnosis, assessment of disease severity, and selection of appropriate diagnostic and therapeutic approaches [37] (see Table 9). Thalassemia, sickle cell disease, membrane disorders, enzymopathies, and congenital dyserythropoietic anemias can share anemia, jaundice, splenomegaly, gallstones, and growth impairment. The diagnostic value of individual features therefore depends on age at onset, transfusion history, family history, hemolytic pattern, and concordance with laboratory findings. Clinical phenotype should be treated as the first layer of evidence in an integrated diagnostic pathway rather than as a stand-alone diagnostic endpoint. However, clinical manifestations such as vaso-occlusive crises, skeletal deformities, membrane abnormalities, oxidative hemolysis, and ineffective erythropoiesis help in the correct clinical diagnosis and direction of further laboratory and molecular investigations [38].

#### 4.1.3. Complications of Inherited Anemias

Table 10 summarizes the major implications of inherited anemias. Long-term morbidity is more than anemia [39]. Complications such as iron overload, persistent hemolysis, cardiovascular and endocrine dysfunction, infections, skeletal deformities, and psychosocial impairment have a great impact on the prognosis and quality of life in patients. These complications are addressed in clinical reviews by Piel et al. [20] (thalassemia review), Agarwal & Rets [2] (hereditary hemolytic anemias), Roy et al. [3] (BSH/EHA Good Practice Paper), and Russo et al. [5] (congenital dyserythro-poietic anemias). Knowledge about these complication patterns is the basis for timely monitoring, preventive measures, and individualized multidisciplinary care to relieve the burden of the illness and to improve long-term prognosis [13].

Inherited anemias continue to be a significant public health problem around the world, and the absence of effective prevention and management techniques contributes to more than 300,000 stillbirths each year affecting children with severe inherited anemias [39].

#### 4.1.4. Differential Clinical Features

Roy et al. [3], Agarwal & Rets [2], and Çiftçiler [40] specifically examine differential clinical aspects of major inherited anemias and provide direct evidence for distinguishing inherited anemias based on age of onset, clinical presentations, hallmark features, and diagnostic clues. Piel et al. [20] described “thalassemia,” Russo et al. [5] congenital dyserythropoietic anemias, and Kaestner et al. [7] inherited membrane diseases. Table 11 shows a comparison between divergent clinical aspects of major hereditary anemias, age at presentation, typical symptoms, and different clinical clues. Although there are some common features across several disorders such as chronic anemia and hemolysis, differences in disease onset, clinical presentation, and hallmark findings such as vaso-occlusive crises, transfusion dependence, membrane abnormalities, oxidative hemolysis, and syndromic features are of immense help for differential diagnosis and subsequent molecular confirmation [20].

### 4.2. Laboratory Diagnosis of Inherited Anemias

Laboratory diagnosis of inherited anemias involves a systematic approach with a battery of hematological, biochemical, morphological, special laboratory, and molecular genetic tests to detect, classify, and characterize hereditary defects of red blood cells [41]. It encompasses routine tests such as complete blood count, red blood cell indices, peripheral blood smear examination, reticulocyte assessment, hemoglobin analysis, specialized functional assays, and molecular genetic testing for accurate diagnosis, differentiation of disease subtypes, assessment of disease severity, support of genotype–phenotype correlation, and guiding personalized therapeutic management [16].

#### 4.2.1. Complete Blood Count (CBC)

The complete blood count (CBC) is an inexpensive and widely available first-line investigation that provides hemoglobin concentration, cell counts, and red-cell indices. Its main diagnostic value is triage: patterns of microcytosis, macrocytosis, anisocytosis, and reticulocytosis narrow the differential diagnosis and determine which specialized tests should follow. However, CBC abnormalities are not disease-specific and may be altered by iron deficiency, inflammation, recent transfusion, age, pregnancy, or mixed nutritional states. CBC parameters should therefore be interpreted in conjunction with iron studies, morphology, hemoglobin analysis, hemolysis markers, and molecular testing when the phenotype remains unresolved [42].

Table 12 shows diagnostic significance of complete blood count (CBC) parameters in the initial assessment of inherited anemias Routine measurements such as hemoglobin, red blood cell count, hematocrit, mean corpuscular volume, mean corpuscular hemoglobin, mean corpuscular hemoglobin concentration, and red cell distribution width can provide useful information on the severity of anemia, erythropoietic activity, red blood cell morphology, and the underlying disease mechanisms. These measures are useful as primary indicators that assist in differential diagnosis and the need for specific laboratory and molecular investigations [43].

#### 4.2.2. Red Blood Cell Indices

Red-cell indices, including MCV, MCH, and MCHC, provide standardized measures of erythrocyte size and hemoglobin content and remain central to morphological anemia classification [44]. Their strength is availability and low cost; their limitation is incomplete specificity. Thalassemia trait and iron-deficiency anemia can produce overlapping microcytic patterns, and discriminant formulas show variable performance across populations, prevalence settings, analyzer platforms, and cutoff definitions. Previous research studies use the indices for different application, among these is the work of Hataysal & K˝orez [45] that developed a machine learning model using routine red blood cell indices to distinguish with good accuracy the β-thalassemia trait, iron deficiency anemia and healthy individuals. Similar to this, Aljedai et al. [46] assessed the effect of recurrent blood donation on red blood cell indices, reticulocyte characteristics and iron metabolism by performing extensive hematological examinations.

Accordingly, red-cell indices are best used as screening and triage variables rather than confirmatory tests [45,47,48].

The diagnostic significance of red blood cell (RBC) indices in inherited anemias is summarized in Table 13. RBC indices (MCV, MCH, MCHC), RDW, and RBC count are helpful in determining red cell size, hemoglobin concentration, membrane integrity, and cell variation. These indices are useful for classification of anemia, identification of membrane abnormalities, assessment of anisocytosis, and distinction of thalassemia from other forms of anemia and are important parts of the first hematological examination [3].

The most common discriminant indices used for differential diagnosis are mathematical calculated hematological formulas based on routine complete blood count (CBC) parameters, mainly red blood cell indices, to differentiate inherited anemias, mainly beta-thalassemia trait from other microcytic anemias such as iron deficiency anemia [47]. Cost-effective screening tools such as Mentzer, England and Fraser, Shine and Lal, Green and King, Srivastava, and RDW indices assist in early differential diagnosis, reduce the need for unnecessary laboratory investigations, and help in the selection of appropriate confirmatory hematological and molecular diagnostic tests [3].

Table 14 demonstrates the commonly used discriminant indices to differentiate thalassemia trait from iron deficiency anemia using routine hematological parameters. Indices such as the Mentzer, Shine and Lal, England and Fraser, Green and King, and RDW Index (RDWI) are basic, inexpensive screening methods that increase diagnosis accuracy before additional laboratory or molecular testing is conducted [48]. Their application supports appropriate clinical decision making and reduces unnecessary confirmatory investigations [2].

#### 4.2.3. Peripheral Blood Film (Peripheral Blood Smear)

Peripheral blood-film examination provides rapid phenotypic information on red-cell shape, size, inclusions, polychromasia, and fragmentation. Its value is greatest when characteristic patterns support a focused differential diagnosis; however, many findings are non-specific and interpretation is influenced by smear quality, staining, observer expertise, transfusion history, and inter-observer variability. Morphology should therefore complement rather than replace quantitative hematology, biochemical assays, hemoglobin analysis, and molecular confirmation [3,49,50].

Table 15 shows the typical peripheral blood smear findings of major hereditary anemias and their diagnostic importance. The characteristic morphological abnormalities such as sickle cells, target cells, spherocytes, elliptocytes, stomatocytes, bite cells, and basophilic stippling reflect the underlying pathophysiologic mechanisms of each disorder. Thus, peripheral blood smear examination continues to be an important first-line diagnostic tool, adding valuable morphological evidence to hematological, biochemical, and molecular investigations for accurate diagnosis and disease classification [7].

Morphological features identification and their diagnostic interpretation are done through the microscopic examination of abnormalities of red blood cells on peripheral blood smear to identify characteristic morphological patterns that help in the diagnosis, classification, differential diagnosis, and severity assessment of inherited anemias guiding further laboratory and molecular investigations [2]. It is known that the identification of a certain morphological aberration [51] can be used to determine the differential diagnosis of some kinds of anemia. The test of peripheral blood smear (PBS) is an important component in the diagnosis of hematological illnesses. Anemia can be detected with PBS. The manual analysis approach is still used, even though it is labor intensive, error prone, and time consuming and requires trained laboratorians.

Table 16 outlines the diagnostic interpretation of distinctive red blood cell morphological features seen on inspection of peripheral blood smears. The specific abnormalities, i.e., sickle cells, target cells, spherocytes, elliptocytes, stomatocytes, bite cells, Heinz bodies, basophilic stippling, nucleated red blood cells, polychromasia, and Howell–Jolly bodies, provide important clues to the underlying pathophysiological mechanisms of inherited anemias. Recognition of these morphological features allows the establishment of differential diagnosis and guides targeted laboratory and molecular investigations for accurate disease characterization [3].

#### 4.2.4. Reticulocyte Count and Hemolytic Biomarkers

Reticulocyte counts and hemolysis biomarkers help distinguish inadequate erythropoietic production from increased red-cell destruction. Absolute or corrected reticulocyte counts are generally more informative than percentages alone, but interpretation must account for anemia severity, recent transfusion, marrow suppression, renal function, and treatment effects. Bilirubin, lactate dehydrogenase, haptoglobin, and related markers provide supportive evidence of hemolysis but are not individually specific. The most reliable interpretation therefore comes from concordant trends across reticulocyte response, biochemical markers, morphology, and disease-specific testing [52]. Supporting this interpretation, Sah and Rao [53] emphasize that corrected reticulocyte counts and the reticulocyte production index help assess marrow response, distinguish reduced production from compensatory erythropoiesis, and monitor treatment effectiveness.

Table 17 lists the main reticulocyte and hemolytic biomarkers utilized for evaluation of hereditary anemias. These biomarkers are important in providing information on the response of the bone marrow, erythropoietic activity, and the presence and severity of hemolysis. Parameters including reticulocyte count, reticulocyte production index, lactate dehydrogenase, unconjugated bilirubin, haptoglobin, plasma-free hemoglobin, and hemoglobinuria all assist in differentiating regenerative and non-regenerative anemia, identifying intravascular hemolysis, and guiding further diagnostic testing and clinical management [3].

Interpretation of reticulocyte count in major hereditary anemias refers to the clinical application of absolute or corrected reticulocyte counts for evaluation of adequacy of bone marrow erythropoietic response and for differentiation of underlying pathophysiological mechanisms of inherited anemias [54]. Increased reticulocyte counts are usually indicative of a compensatory erythropoiesis response to chronic hemolysis, as in sickle cell disease, hereditary spherocytosis, or glucose-6-phosphate dehydrogenase deficiency, while decreased or inappropriately normal reticulocyte counts are indicative of ineffective erythropoiesis or bone marrow failure, as in β-thalassemia major, congenital dyserythropoietic anemia, and Diamond–Blackfan anemia. Hence, the reticulocyte count interpretation is important for illness classification, assessment of disease severity, monitoring of the response to treatment, and guiding further hematological and genetic studies [20].

Table 18 shows the expected reticulocyte responses in the major inherited anemias. These responses are of diagnostic importance in evaluating bone marrow function. Increased reticulocyte numbers are generally adequate for the amount of hemolysis, and low or inappropriately normal counts are consistent with ineffective erythropoiesis or bone marrow failure. Thus, analysis of disease-specific reticulocyte patterns gives important clues to the underlying pathophysiology and helps the differential diagnosis and clinical management of inherited anemias [13].

#### 4.2.5. Hemoglobin Analysis and Electrophoretic Techniques

Hemoglobin analysis uses complementary separation techniques, particularly electrophoresis, cation-exchange high-performance liquid chromatography (HPLC), capillary electrophoresis, and isoelectric focusing, to identify and quantify normal and abnormal hemoglobin fractions [55,56,57,58]. HPLC offers high-throughput quantification and reproducible retention patterns, while capillary electrophoresis provides high-resolution automated separation. Neither method is infallible: co-elution, uncommon variants, recent transfusion, compound heterozygosity, and platform-specific patterns can generate ambiguous results. Discordant or clinically unexpected profiles should therefore be confirmed using an orthogonal phenotypic method and/or molecular analysis rather than interpreted from a single platform.

Table 19 demonstrates the comparison of key hemoglobin analysis techniques used for the diagnosis of inherited anemias: principles, clinical applications, strengths, and limits. Hemoglobin variations can be detected and characterized utilizing complimentary methodologies such as hemoglobin electrophoresis, high-performance liquid chromatography, capillary electrophoresis, isoelectric focusing, and mass spectrometry. The choice of the approach improves the diagnostic accuracy and allows the identification of hemoglobinopathies, enabling the successful clinical care and the subsequent molecular confirmation, if needed [16].

Hemoglobin analysis techniques such as high-performance liquid chromatography (HPLC), capillary electrophoresis (CE), hemoglobin electrophoresis, and isoelectric focusing (IEF) provide characteristic qualitative and quantitative profiles of hemoglobin fractions [59]. These profiles are characterized by the relative proportions of normal hemoglobins (HbA, HbA_2_, and HbF) and abnormal hemoglobin variants (e.g., HbS, HbC, HbE, and HbD), supporting the diagnosis and classification of inherited hemoglobin disorders. Accurate characterization of the underlying hemoglobin disorder is also important for determining eligibility for disease-specific advanced therapies; for example, betibeglogene autotemcel (Zynteglo) is an FDA-approved gene therapy for patients with β-thalassemia who require regular red blood cell transfusions [60]. These specific profiles are also useful for interpretation of illness severity, genetic counseling, therapeutic decision, and validation by molecular genetic testing [3].

Table 20 shows the typical hemoglobin patterns in common inherited hemoglobin disorders. Differences in the relative amounts of HbA, HbA_2_, and HbF help in identifying the disease. Characteristic patterns, such as increased HbA_2_ in β-thalassemia trait, predominant HbF in β-thalassemia major, predominant HbS in sickle cell disease, and HbC or HbE variants, provide important diagnostic clues. Interpretation of these hemoglobin profiles together with clinical findings and molecular testing can improve the diagnosis and classification of hemoglobinopathies [10]. Accurate disease classification is also increasingly important for treatment selection. For example, the U.S. FDA indicates lovotibeglogene autotemcel (Lyfgenia^®^) for patients aged 12 years or older with sickle cell disease and a history of vaso-occlusive events [61]. Exagamglogene autotemcel (Casgevy^®^) is indicated for defined patients with sickle cell disease with recurrent vaso-occlusive crises and transfusion-dependent β-thalassemia [62]. Thus, accurate differentiation of inherited hemoglobin disorders has implications not only for diagnosis but also for determining eligibility for disease-specific advanced therapies. Extending molecular diagnostic strategies, Onay et al. [63] demonstrate how deep-intronic sequencing and RNA analysis resolved an undiagnosed Pompe case, illustrating a complementary approach to variant detection rather than direct evidence for inherited anemia.

#### 4.2.6. Specialized Laboratory Assays

Specialized laboratory assays complement routine hematological investigations when the initial clinical and laboratory findings do not establish a definitive diagnosis. Depending on the suspected inherited anemia, these investigations may include red blood cell membrane studies such as the eosin-5′-maleimide (EMA) binding test, osmotic fragility testing, and osmotic gradient ektacytometry and red blood cell enzyme assays such as glucose-6-phosphate dehydrogenase or pyruvate kinase deficiency [12]. The studies should be evaluated in the light of the suspected disease and together with the clinical and routine hematological findings. Molecular testing can be helpful to provide extra information in instances that are aberrant, overlapping, or unresolved when identification of the underlying genetic issue is clinically relevant [5,6]. They provide definite evidence of membrane, enzyme, and hemoglobin abnormality, allow differential diagnosis, assist in genotype-phenotype correlation, and guide molecular confirmation, disease monitoring, and personalized clinical management of inherited anemias [2,50].

Table 21 provides a summary of specific laboratory assays used in the diagnosis of hereditary anemias, including their diagnostic aims, clinical uses, advantages, and limitations. These assays are complementary to regular hematological investigations and allow for detailed assessment of red blood cell membrane integrity, enzyme activity, erythrocyte deformability, bone marrow morphology, and iron metabolism. The proper selection and combination of these special tests improve diagnostic accuracy, allow for accurate disease classification, and support personalized patient management, especially in complex or atypical cases [7].

### 4.3. Molecular Genetic Diagnosis

Molecular testing has improved etiological resolution in phenotypically overlapping and genetically heterogeneous inherited anemias. Targeted PCR and Sanger sequencing remain efficient for known variants, whereas gene panels, WES, WGS, and third-generation sequencing broaden detection of rare, structural, and complex variants [2,3,5,14,18]. Higher analytical sensitivity does not automatically translate into greater clinical certainty: variants of uncertain significance, incomplete penetrance, coverage gaps, difficult structural regions, ancestry-related reference limitations, and imperfect genotype-phenotype correlation can complicate interpretation. Molecular results are therefore most useful when reconciled with clinical phenotype and laboratory findings in a multidisciplinary diagnostic process.

Table 22 provides a comparison of the main molecular diagnostic methods applied in the study of hereditary anemias, including their detection capabilities, clinical applications, benefits, and limitations. Although conventional approaches, such as PCR, Sanger sequencing, and MLPA, are still useful for targeted mutation analysis, NGS, WES, WGS, and third-generation sequencing enable a full genome evaluation with a greater diagnostic yield. The combination of these complementary techniques allows clinicians to select the most appropriate molecular approach for the suspected disorder, thus improving diagnostic accuracy, enabling personalized patient care, and contributing to the progress of precision medicine in inherited hematology [2].

### 4.4. Contemporary Therapeutic Approaches

The current management of the condition involves blood transfusion, iron chelation, pharmacological medications, hematopoietic stem cell transplantation, gene therapy, and genome editing to lessen the consequences of the disease and increase survival and quality of life [64]. The development of individualized therapeutic strategies, focusing both on the disease manifestations and the genetic defects underlying them, has been made possible by advances in molecular medicine, leading to better long-term clinical outcomes for patients [20].

Table 23 outlines the major components of supportive therapy for hereditary anemias. These interventions include red blood cell transfusion, folic acid supplementation, vaccination, antibiotic prophylaxis, clinical monitoring, MRI T2* assessment, psychological and nutritional support, and patient education, which are important in controlling symptoms, preventing complications, and improving quality of life. Supportive therapies do not address the underlying genetic abnormalities but are critical for complete illness management and long-term patient care [3].

#### 4.4.1. Blood Transfusion Therapy

Adequate hemoglobin levels, improved tissue oxygenation, suppression of ineffective erythropoiesis, and prevention of life-threatening consequences like stroke and acute chest syndrome are necessary and can be achieved through regular or tailored transfusion techniques [65]. Long-term therapy of transfusion-dependent illnesses still relies on appropriate transfusion protocols with close monitoring for transfusion-associated consequences [13].

Table 24 presents the summary of the main clinical indications and optimal transfusion techniques for transfusion-dependent disorders. For disorders such as ß-thalassemia major and Diamond–Blackfan anemia, regular simple transfusions are recommended, while in sickle cell disease, exchange transfusion is often used for the prevention or treatment of severe consequences. Congenital dyserythropoietic anemia and pyruvate kinase deficiency also involve individualized or symptom-directed transfusion strategies as a result of heterogeneity in disease severity, clinical presentation, and therapeutic goals [13].

#### 4.4.2. Iron Chelation Therapy

Iron chelation therapy is the administration of iron-binding drugs to eliminate excess body iron due to continuous transfusion therapy or increased iron absorption in hereditary anemias [66]. Iron chelation is important for improved long-term results and supporting individual disease treatment by lowering the buildup of iron in tissues and preventing organ damage [35].

Table 25 presents the available iron chelators at present for the therapy of transfusion-associated iron excess. Deferoxamine, deferiprone, and deferasirox differ in their mode of administration, efficacy, and safety profiles, allowing therapy to be tailored to the individual patient needs. Strict selection and monitoring of chelation therapy are critical to minimize iron-induced organ damage, improve compliance, and optimize long-term clinical results in transfusion-dependent hereditary anemias [35].

#### 4.4.3. Disease-Modifying Pharmacological Therapy

Disease-modifying pharmacotherapy increasingly targets mechanisms specific to individual inherited anemias. Hydroxyurea increases fetal hemoglobin in sickle cell disease; luspatercept promotes erythroid maturation in selected patients with beta-thalassemia; and mitapivat activates red-cell pyruvate kinase in pyruvate kinase deficiency. These therapies should not be generalized across inherited anemias because indications, age groups, regulatory status, and benefit-risk profiles are disease- and jurisdiction-specific. Voxelotor is retained in Table 26 only as a historical example: Oxbryta was voluntarily withdrawn from the market in September 2024 after postmarketing evidence raised safety concerns, and the FDA advised clinicians to stop prescribing it [67].

Table 26 therefore distinguishes currently used disease-specific therapies from agents whose clinical status has changed. Effectiveness should be interpreted against pivotal trials and contemporary disease-specific guidance, with explicit attention to eligibility criteria, adverse effects, durability of response, and post-authorization safety surveillance. Regulatory status is particularly important when interpreting disease-modifying therapies because authorization may change as confirmatory or post-marketing evidence emerges. Crizanlizumab (Adakveo^®^), for example, received conditional EU marketing authorization in 2020, but this authorization was revoked by the European Commission on 3 August 2023 after the confirmatory STAND study failed to establish the anticipated clinical benefit. Similarly, voxelotor (Oxbryta^®^) was voluntarily withdrawn from the U.S. market in September 2024 following emerging safety concerns. Accordingly, Table 26 reports regulatory status together with jurisdiction and relevant dates rather than treating historical authorization as evidence of current clinical availability.

#### 4.4.4. Hematopoietic Stem Cell Transplantation (HSCT)

Allogeneic hematopoietic stem-cell transplantation (HSCT) can provide definitive or potentially curative treatment for selected severe inherited hematological disorders by replacing ineffective or genetically abnormal hematopoiesis with donor-derived hematopoiesis [68]. Its benefit must be balanced against graft-versus-host disease, graft failure, infection, infertility, organ toxicity, and transplant-related mortality. Outcomes vary with diagnosis, age, donor type, conditioning regimen, pre-existing organ damage, and center expertise. Current guidance therefore supports individualized selection rather than describing HSCT as uniformly curative across all inherited anemias. Alongside transplantation, genome editing offers another therapeutic direction. Aljabali et al. [69] review CRISPR-Cas9 principles and delivery advances, providing a technological foundation for understanding gene-editing approaches discussed in inherited anemia management.

Table 27 summarizes principal clinical applications of HSCT. The strongest established evidence concerns selected patients with transfusion-dependent beta-thalassemia, sickle cell disease, and inherited bone-marrow-failure syndromes; evidence for rarer inherited anemias is more limited and often based on small series or specialist-center experience.

#### 4.4.5. Gene Therapy

Gene-based therapy has moved from experimental development into regulated clinical use for selected hemoglobinopathies, but authorization is product-, indication-, age-, and jurisdiction-specific. As of August 2026, the U.S. FDA lists betibeglogene autotemcel (Zynteglo) for adult and pediatric patients with beta-thalassemia requiring regular red-cell transfusions; lovotibeglogene autotemcel (Lyfgenia) for patients aged 12 years or older with sickle cell disease and a history of vaso-occlusive events; and exagamglogene autotemcel (Casgevy) for patients aged 2 years or older with recurrent vaso-occlusive sickle cell disease or transfusion-dependent beta-thalassemia [60,61,62]. These approvals do not establish universal applicability, indefinite durability, or absence of late toxicity.

Table 28 distinguishes authorized gene-based products from investigational approaches. Regulatory status also differs between jurisdictions; for example, European authorization criteria for Casgevy remain indication-specific and should not be assumed to mirror current U.S. age eligibility. Long-term follow-up remains essential because conditioning-related toxicity, fertility effects, clonal complications, malignancy risk, and durability of benefit may emerge over time.

#### 4.4.6. Genome Editing (CRISPR-Cas9 and Emerging Technologies)

Genome editing should be divided into established clinical evidence and emerging experimental approaches. Ex vivo CRISPR/Cas9 editing has regulatory authorization for defined sickle cell disease and transfusion-dependent beta-thalassemia populations through exagamglogene autotemcel. In contrast, most base-editing, prime-editing, epigenome-editing, and other next-generation strategies remain investigational. Claims of “cure,” low off-target risk, or long-term durability are therefore premature for experimental platforms unless supported by adequate clinical follow-up. The appropriate description is potentially curative or associated with sustained benefit during the currently available follow-up.

Table 29 summarizes this distinction between clinically authorized CRISPR/Cas9-based therapy and emerging genome-editing platforms. Comparative effectiveness against HSCT and long-term disease-modifying therapy remains incompletely defined, and implementation is constrained by myeloablative conditioning, manufacturing complexity, specialized infrastructure, high cost, and requirements for long-term surveillance.

## 5. The Principal Findings

The literature search and selection process identified 69 publications for the final evidence synthesis after deduplication, title and abstract screening, and full-text eligibility assessment (Table 30). The evidence base included original research, systematic reviews and meta-analyses, relevant narrative reviews, clinical practice guidelines, consensus statements, and selected landmark publications addressing clinical, laboratory, molecular, diagnostic, and therapeutic aspects of inherited anemias.

Eleven sources were mostly concerned with the clinical aspect of inherited anemias. They talked about the presentation of disease, epidemiology, comorbidities, transfusion-dependent thalassemia, iron chelation, clinical treatment, and potentially curative drugs. Such studies support the first stage of the framework and illustrate that clinical suspicion of an inherited anemia is characterized by patient history, symptoms, physical findings, severity of illness, transfusion requirements, and consequences.

Of the three basic domains, clinical medicine, however, was the least represented. This implies that the review is more focused on diagnosis and molecular characterization rather than longitudinal clinical outcomes, quality of life, therapy adherence, and patient-reported outcomes. Laboratory hematology was the most common area with 21 mentions. The evidence comprised “Complete blood count and red blood cell indices,” “Discriminant indices for thalassemia and iron deficiency anemia,” “Peripheral blood smear morphology,” “Reticulocyte assessment,” “Hemolytic biomarkers,” “Hemoglobin electrophoresis,” “High-performance liquid chromatography,” “Capillary electrophoresis,” “Specialized membrane and enzyme testing,” and “Machine-learning-assisted analysis of hematological data.”

Laboratory hematology appears to offer the major phenotypic basis for classification and informs additional disease-specific testing. Routine and specialized assays serve to differentiate membrane diseases, enzymopathies, thalassemias, hemoglobin variations, and other inherited red-cell abnormalities, with molecular testing utilized judiciously in cases where conventional findings are ambiguous, unusual, or clinically inadequate.

Sixteen dealt mainly with molecular genetics. Key subjects discussed were next-generation sequencing, third-generation sequencing, gene panels, prenatal diagnostics, cell-free fetal DNA, genomic characterization, genotype–phenotype connections, and CRISPR-related therapeutic discoveries.

The paradigm is supported by molecular data showing that genetic testing can validate equivocal laboratory findings, detect rare variants, discriminate overlapping phenotypes, elucidate inheritance patterns, and give information for family screening and genetic counseling. They are especially effective in patients with equivocal or unusual clinical and laboratory data.

Together, our findings indicate an integrated disease- and context-dependent diagnostic route rather than a universal sequential model. The diagnostic context and care needs are defined by clinical assessment, the phenotype is characterized by laboratory hematology, and the differential diagnosis is focused, with molecular testing integrated when indicated to resolve uncertain or complex cases, clarify inheritance, support genetic counseling, or inform disease-specific management.

Hence the framework permits diagnosis to be made at multiple points depending on the disease and evidence available. In some instances, characteristic clinical, hematologic, biochemical, or functional findings are adequate for reliable diagnosis; in others, molecular testing may be required to elucidate an aberrant or overlapping phenotype. Genetic data have to be interpreted according to clinical and laboratory evidence, as the diagnostic certainty may be limited by variations of unknown importance and insufficient genotype–phenotype correlation.

The suggested approach is strongly supported by the literature examined, notably the combination of laboratory hematology and molecular genetics. Diagnosis is mostly based on laboratory findings. Molecular techniques are used for validation and characterization of complicated hereditary anemias. Clinical medicine provides the patient context to interpret these findings and apply them to individualized therapy. Together, the three areas provide a patient-centered precision medicine route that may improve accuracy of diagnosis, classification of diseases, selection of treatment, genetic counseling, and long-term patient outcomes.

All 69 papers included in the final evidence base met the pre-specified criteria for eligibility and relevance. Of these, 48 corresponded directly to one of the three core domains (clinical medicine, laboratory hematology, or molecular genetics), and 21 presented appropriate cross-cutting or multidomain evidence applicable to more than one component of the framework. Publications that were cited only for broad physiological background, historical context, explanation of methods, or regulatory information were considered contextual references and were not included in the 69 evidence-based publications.

All 69 papers included in the final evidence synthesis were subjected to a further design-sensitive methodological quality evaluation. Eligible systematic reviews were appraised using AMSTAR 2, and design-appropriate criteria were employed for additional evidence categories. That appraisal was part of the evidence weighting phase, not a further eligibility check. The synthesis incorporated source-level judgments to give more interpretative weight to methodologically stronger and more directly relevant evidence and to express conclusions based on methodologically limited or indirect evidence more cautiously.

The evidence framework illustrates the synergistic roles of clinical assessment, laboratory hematology, and molecular genetics in the evaluation of hereditary anemias (see Table 31). Laboratory examinations provide the main phenotypic and hematological bases for disease characterization. Molecular methods can confirm and resolve genetically heterogeneous and diagnostically ambiguous situations. Clinical evidence offers the context for interpreting laboratory and molecular findings when considering illness presentation, severity, consequences, and management. Evidence that cuts across these domains further indicates integration throughout the domains.

## 6. Critical Synthesis and Discussion

### 6.1. Integrated Interpretation of the Evidence

The central finding of this review is that inherited-anemia diagnosis is most reliable when clinical phenotype, laboratory hematology, and molecular genetics are interpreted as complementary layers of evidence. Clinical assessment defines the diagnostic context but lacks specificity; routine hematology is accessible and phenotypically informative but cannot resolve all overlapping disorders; and molecular testing improves etiological resolution but can generate variants of uncertain significance, coverage gaps, and genotype-phenotype ambiguity. The framework should therefore be interpreted as an evidence-informed escalation pathway rather than as a claim that one modality replaces another.

### 6.2. Diagnostic Performance, Comparative Value, and Limitations

The reviewed evidence favors complementary diagnostic testing rather than reliance on a single technology. CBC and red-cell indices are useful for triage but have variable specificity across populations; blood-film interpretation is rapid but observer-dependent; HPLC and capillary electrophoresis provide high-throughput hemoglobin characterization but may show co-elution or platform-specific ambiguities; and molecular methods improve resolution of complex cases but require careful variant interpretation. Diagnostic performance therefore depends on disease prevalence, pre-test probability, patient ancestry, transfusion history, assay platform, reference standards, and availability of confirmatory testing. Direct head-to-head diagnostic-accuracy studies remain limited for several rare inherited anemias.

### 6.3. Therapeutic Evidence, Uncertainty, and Comparative Effectiveness

Therapeutic evidence is strongest for established transfusion and chelation strategies, selected disease-modifying agents, HSCT in defined high-risk disorders, and recently authorized gene-based therapies for specific hemoglobinopathies. However, regulatory approval should not be equated with universal applicability, proven lifetime durability, or absence of late toxicity. Gene addition and CRISPR-based editing require conditioning and specialized infrastructure, while HSCT carries donor-, conditioning-, and graft-related risks. Direct comparative evidence between HSCT and gene-based therapy remains limited, and long-term surveillance is essential for clonal complications, malignancy, fertility effects, organ toxicity, and durability of benefit.

### 6.4. Implementation Barriers, Evidence Gaps, and Novel Contribution

Implementation remains uneven because advanced molecular testing and gene-based therapies require substantial laboratory infrastructure, bioinformatics capacity, specialized clinical expertise, genetic counseling, long-term follow-up, and financial resources. These constraints are especially important in regions with a high burden of hemoglobinopathies. Future research should prioritize ancestry-diverse genomic reference datasets, prospective comparative diagnostic studies, cost-effectiveness of tiered testing algorithms, standardized reporting of variants of uncertain significance, long-term comparative effectiveness of HSCT and gene-based therapies, and implementation strategies for resource-limited settings. The novel contribution of this review is therefore the critical integration of clinical, phenotypic-laboratory, and molecular evidence into a patient-centered precision-medicine framework that makes the strengths, limitations, uncertainties, and escalation points of each diagnostic and therapeutic domain explicit.

To substantiate this integrated contribution, the key evidence that directly supports the diagnostic approach is summarized below. The studies identified highlight how clinical assessment, specialist laboratory investigation, and genetic techniques provide complementary data across hereditary anemia subtypes. Phenotypic differentiation is supported by clinical and laboratory investigations, while molecular investigations show the increasing influence of NGS, targeted genetic testing, and structural-variant detection for diagnostically challenging cases: hemoglobinopathies, membrane disorders, and enzymopathies. These findings support the proposed tiered precision-medicine system but also underscore the need for future comparative diagnostic research, greater genetic representation, consistent interpretation, and enhanced application in resource-limited situations.

A number of papers further contribute to the suggested comprehensive diagnostic paradigm. This is represented in Table 32. Agarwal and Rets [2] review the current molecular diagnosis of hereditary hemolytic anemias, while Roy et al. [3] present good-practice evidence for the application of next-generation sequencing for rare inherited anemias. Russo et al. [5] extend this basis with genetics and genomics techniques, whereas Steinberg-Shemer and Tamary [6] show the diagnostic and therapeutic importance of NGS in congenital anemias.

In addition to these contributions, Kaestner et al. [7] review emerging diagnostic tools for red-cell disorders, while Suhaimi et al. [14] and Achour et al. [16] show the expanding use of NGS for thalassemia and hemoglobinopathy screening and diagnosis. Collectively, these studies provide particularly important support for the combination of phenotypic laboratory examination with targeted and genomic testing in diagnostically challenging hereditary anemias.

## 7. Conclusions

Inherited anemias comprise heterogeneous genetic disorders with overlapping clinical phenotypes, laboratory patterns, and molecular mechanisms. This review provides qualitative support for an integrated patient-centered precision-medicine framework in which clinical assessment establishes diagnostic context, laboratory hematology characterizes the phenotype, and molecular testing resolves selected uncertain or complex cases. The evidence does not support replacing conventional hematology with genomics; rather, diagnostic accuracy is greatest when methods are used sequentially and complementarily. Confidence is strongest for established laboratory workflows and selected disease-specific therapies, while comparative effectiveness, long-term durability and safety of emerging gene-based interventions, and equitable implementation remain important uncertainties. The framework should therefore be regarded as an evidence-informed integrative model rather than a formally validated clinical pathway.

## Figures and Tables

**Table 1:** Clinical characteristics of major inherited anemias.

Disease	Primary Genetic Defect	Major Clinical Manifestations	Common Laboratory Findings	Major Complications
Thalassemia Major	Reduced or absent globin chain synthesis	Severe anemia, jaundice, hepatosplenomegaly, growth retardation	Low Hb, microcytosis, elevated HbF	Iron overload, skeletal deformities
Thalassemia Intermedia	Partial globin chain deficiency	Moderate anemia, splenomegaly	Mild-to-moderate microcytic anemia	Gallstones, osteoporosis
Thalassemia Trait	Heterozygous globin mutation	Usually asymptomatic	Mild microcytic anemia	Rare complications
Sickle Cell Disease	β-globin mutation (HbS)	Pain crises, chronic hemolysis, jaundice	Sickled erythrocytes, elevated reticulocytes	Stroke, splenic sequestration

**Table 2:** The databases used for the research.

Database	Primary Coverage
PubMed/MEDLINE	Biomedical sciences, clinical medicine, genetics, laboratory medicine
Scopus	Multidisciplinary scientific literature
Web of Science	High-impact multidisciplinary journals
Embase	Biomedical research, pharmacology, laboratory medicine
Google Scholar	Scholarly literature and gray literature
Cochrane Library	Evidence-based healthcare
Reference Lists of Retrieved Articles	Bibliographic citations

**Table 3:** Summary of the database-specific search approach, limits, and search period.

Database/Source	Search Approach	Limits	Search Date
PubMed/MEDLINE	Database-specific controlled vocabulary and free-text Boolean search	Human; English; principally 2015–2026	6-May-2026 to 31-July-2026
Scopus	TITLE-ABS-KEY Boolean searches across predefined inherited-anemia concept groups	Article/review; English; 2015–2026	6-May-2026 to 31-July-2026
Web of Science	Topic-field Boolean searches adapted to Web of Science syntax	English; principally 2015–2026	6-May-2026 to 31-July-2026
Embase	Controlled vocabulary and free-text Boolean search adapted to Embase	Human; English; principally 2015–2026	6-May-2026 to 31-July-2026
Google Scholar	Multiple predefined inherited-anemia and diagnostic concept searches	Principally 2015–2026	6-May-2026 to 31-July-2026
Cochrane Library	Boolean search adapted to Cochrane Search Manager	Relevant human evidence; principally 2015–2026	6-May-2026 to 31-July-2026
Reference lists	Manual backward reference checking of eligible and landmark publications	Eligibility criteria applied	6-May-2026 to 31-July-2026

**Table 4:** Inclusion and exclusion criteria.

Inclusion Criteria	Exclusion Criteria
Studies investigating inherited or hereditary anemia disorders	Studies on acquired anemia without a genetic basis
Original research articles	Animal or in vitro studies only
Systematic reviews and meta-analyses	Editorials, letters, opinions, or commentaries
Narrative review articles	Conference abstracts lacking sufficient data
Clinical practice guidelines and consensus statements	Case reports (unless describing a unique or novel inherited disorder)
Studies involving laboratory diagnosis, molecular diagnosis, genetics, or clinical management	Duplicate publications
Human population studies	Non-English publications
Full-text articles	Articles with unavailable full text
Publications between 2015 and 2026 (with landmark earlier studies included where necessary)	Studies unrelated to inherited anemia or hereditary red blood cell disorders

**Table 5:** Distribution of displayed search hits during the exploratory literature search.

Source/Search Set	Displayed Results	Percentage of Displayed Aggregate	Status
PubMed/MEDLINE	10,916	3.91	Actual database results
Web of Science	24,576	8.80	Actual database results
Scopus: nine separate categories	74,448	26.66	Sum contains overlaps between categories
Google Scholar: eight searches	169,286	60.63	Approximate exploratory scholarly records
Displayed aggregate	279,226	100.00	Not the number of unique records

**Table 6:** Study selection criteria.

Selection Component	Description	Purpose
Eligible Studies	Studies fulfilling all inclusion criteria	Ensure relevance to the review objective
Publication Period	2015–2026 (primary focus)	Capture recent scientific developments
Landmark Studies	Seminal publications published before 2015	Provide historical and theoretical foundations
Study Types	Original research, systematic reviews, meta-analyses, narrative reviews, clinical guidelines, consensus papers	Obtain comprehensive scientific evidence
Research Focus	Clinical, laboratory, molecular, diagnostic, genetic, and therapeutic aspects	Cover all dimensions of inherited anemia
Population	Human studies	Improve clinical applicability

**Table 7:** Evidence synthesis framework.

Evidence Component	Method of Synthesis	Expected Outcome
Clinical Evidence	Comparative narrative synthesis	Summary of clinical manifestations and disease characteristics
Laboratory Evidence	Comparative analysis of diagnostic methods	Identification of strengths and limitations of laboratory investigations
Molecular Evidence	Thematic synthesis	Evaluation of molecular diagnostic technologies and genomic advances
Historical Evidence	Integration of landmark studies	Explanation of the evolution of inherited anemia diagnosis
Recent Evidence	Comparative review of studies (2015–2026)	Identification of current trends and emerging technologies
Overall Evidence	Integrated narrative synthesis	Balanced evidence-based interpretation supporting clinical practice

**Table 8:** Criteria for assessing confidence in evidence supporting the inherited anemia conceptual framework.

Confidence	Methodological Quality	Consistency	Directness	Interpretation
High	Predominantly high-quality/robust evidence	Findings broadly consistent across multiple independent sources	Directly addresses inherited anemia and framework component	Strong confidence in narrative conclusion
Moderate	Generally acceptable quality with some limitations	Mostly consistent, but some heterogeneity exists	Mainly direct evidence	Reasonable confidence; cautious interpretation warranted
Limited	Important methodological limitations or sparse evidence	Mixed, conflicting, or insufficient findings	Indirect or peripheral evidence	Conclusion considered preliminary or uncertain

**Table 9:** Comparative disease-specific clinical manifestations of major inherited anemias.

Disease Category	Hallmark Clinical Manifestations	Distinguishing Features
Hemoglobinopathies	Chronic hemolytic anemia, jaundice, recurrent pain crises, acute chest syndrome, stroke	Vaso-occlusive crises and multiorgan ischemia
Thalassemia Syndromes	Severe anemia, growth retardation, hepatosplenomegaly, skeletal deformities, iron overload	Bone marrow expansion and transfusion dependence
RBC Membrane Disorders	Chronic hemolysis, jaundice, splenomegaly, pigment gallstones	Spherocytes or elliptocytes; splenic sequestration
RBC Enzyme Disorders	Episodic or chronic hemolysis, jaundice, dark urine, fatigue	Oxidative-triggered hemolysis (G6PD) or chronic ATP deficiency (PK deficiency)
Congenital Dyserythropoietic Anemias	Chronic anemia, ineffective erythropoiesis, hepatosplenomegaly, iron overload	Bone marrow dyserythropoiesis and low reticulocyte response
Other Rare Inherited Anemias	Congenital anomalies, bone marrow failure, recurrent infections, endocrine abnormalities	Multisystem involvement and syndromic presentation

**Table 10:** Major complications of inherited anemias.

Complication	Primary Cause	Major Clinical Consequences	Frequently Associated Disorders
Iron overload	Chronic transfusion and increased intestinal iron absorption	Cardiomyopathy, liver fibrosis, diabetes, endocrine dysfunction	Thalassemia, CDA, transfusion-dependent anemias
Chronic hemolysis	Premature erythrocyte destruction	Jaundice, gallstones, pulmonary hypertension, vascular injury	Sickle cell disease, HS, PK deficiency
Cardiovascular complications	Chronic anemia and myocardial iron deposition	Heart failure, arrhythmias, ventricular dysfunction	Thalassemia, sickle cell disease
Endocrine dysfunction	Iron deposition in endocrine glands	Delayed puberty, infertility, diabetes, hypothyroidism	Thalassemia, CDA
Infections	Functional/asurgical asplenia and immunocompromise	Severe bacterial infections, sepsis	Sickle cell disease, post-splenectomy HS
Growth and skeletal abnormalities	Chronic anemia, marrow expansion, endocrine abnormalities	Growth retardation, osteoporosis, skeletal deformities	Thalassemia, DBA, CDA
Psychosocial complications	Chronic disease burden	Anxiety, depression, reduced quality of life	All inherited anemias

**Table 11:** Differential clinical features of major inherited anemias.

Disease Category	Typical Age at Presentation	Characteristic Clinical Features	Distinguishing Clues
Hemoglobinopathies (Sickle Cell Disease)	Infancy to early childhood	Chronic hemolysis, recurrent painful crises, acute chest syndrome, stroke	Vaso-occlusive episodes and functional asplenia
Thalassemia Syndromes	Infancy (major); adulthood (trait)	Severe anemia, growth retardation, skeletal deformities, iron overload	Bone marrow expansion and transfusion dependence
RBC Membrane Disorders	Childhood to adulthood	Chronic jaundice, splenomegaly, pigment gallstones	Spherocytes or elliptocytes with chronic extravascular hemolysis
RBC Enzyme Disorders	Neonatal period to adulthood	Episodic or chronic hemolysis, dark urine, neonatal jaundice	Oxidative trigger (G6PD) or chronic nonspherocytic hemolysis (PK deficiency)
Congenital Dyserythropoietic Anemias	Childhood	Chronic anemia, ineffective erythropoiesis, iron overload	Low reticulocyte response despite significant anemia
Rare Inherited Anemias	Infancy or childhood	Bone marrow failure, congenital anomalies, endocrine abnormalities	Syndromic presentation with multisystem involvement

**Table 12:** Diagnostic significance of complete blood count parameters in inherited anemias.

CBC Parameter	Diagnostic Significance	Frequently Associated Disorders
Hemoglobin (Hb)	Determines anemia severity	All inherited anemias
RBC Count	Assesses erythropoietic response	Increased in thalassemia trait; reduced in marrow failure
Hematocrit (HCT)	Reflects overall erythrocyte volume	General indicator of anemia severity
Mean Corpuscular Volume (MCV)	Classifies anemia as microcytic, normocytic, or macrocytic	Thalassemia (microcytic); DBA/CDA (macrocytic); HS/SCD (normocytic)
Mean Corpuscular Hemoglobin (MCH)	Indicates hemoglobin content per erythrocyte	Reduced in thalassemia
Mean Corpuscular Hemoglobin Concentration (MCHC)	Identifies erythrocyte dehydration and membrane loss	Elevated in hereditary spherocytosis
Red Cell Distribution Width (RDW)	Measures erythrocyte size variation	Elevated in hemolysis and ineffective erythropoiesis

**Table 13:** Diagnostic significance of red blood cell indices in inherited anemias.

RBC Parameter	Clinical Interpretation	Typical Findings in Inherited Anemias	Diagnostic Value
MCV	Average erythrocyte size	↓ Thalassemia; ↑ DBA/CDA; Normal HS, PK deficiency	Classifies anemia
MCH	Average hemoglobin per erythrocyte	↓ Thalassemia; Usually normal in membrane disorders	Assesses hypochromia
MCHC	Hemoglobin concentration within erythrocytes	↑ Hereditary spherocytosis; ↓ Thalassemia	Suggests membrane defects
RDW	Variation in erythrocyte size	↑ Hemolysis, CDA; Often mildly elevated in thalassemia trait	Assesses anisocytosis
RBC Count	Number of circulating erythrocytes	Often ↑ in thalassemia trait despite anemia	Helps distinguish thalassemia from iron deficiency

**Table 14:** Common discriminant indices used in differential diagnosis.

Index	Formula	Interpretation
Mentzer Index	MCV ÷ RBC count	<13 suggests thalassemia trait; >13 suggests iron deficiency anemia
Shine and Lal Index	MCV^2^ × MCH ÷ 100	Lower values favor thalassemia trait
England and Fraser Index	MCV—RBC—(5 × Hb)—3.4	Negative values favor thalassemia
Green and King Index	(MCV^2^ × RDW) ÷ (Hb × 100)	Used to differentiate thalassemia from iron deficiency
RDW Index (RDWI)	(MCV × RDW) ÷ RBC	Higher values generally indicate iron deficiency anemia

**Table 15:** Characteristic peripheral blood smear findings in major inherited anemias.

Inherited Anemia	Characteristic Peripheral Blood Smear Findings	Diagnostic Significance
Sickle Cell Disease	Sickle cells, target cells, Howell–Jolly bodies, nucleated RBCs, polychromasia	Indicates HbS polymerization and functional asplenia
Thalassemia Syndromes	Microcytosis, hypochromia, target cells, anisopoikilocytosis, basophilic stippling, nucleated RBCs	Suggests defective globin synthesis and ineffective erythropoiesis
Hereditary Spherocytosis	Spherocytes, polychromasia	Supports membrane protein defects; correlate with EMA test
Hereditary Elliptocytosis	Elliptocytes, ovalocytes	Suggests spectrin or protein 4.1 abnormalities
Hereditary Stomatocytosis	Stomatocytes	Indicates altered membrane ion permeability
G6PD Deficiency	Bite cells, blister cells, Heinz bodies (supravital stain), polychromasia	Indicates oxidative hemolysis
Pyruvate Kinase Deficiency	Polychromasia, echinocytes, anisopoikilocytosis	Reflects chronic nonspherocytic hemolysis
Congenital Dyserythropoietic Anemia	Anisopoikilocytosis, nucleated RBCs, basophilic stippling	Suggests ineffective erythropoiesis; bone marrow examination required
Congenital Sideroblastic Anemia	Microcytosis, hypochromia, anisopoikilocytosis, basophilic stippling	Requires bone marrow confirmation of ring sideroblasts

**Table 16:** Morphological features and their diagnostic interpretation.

Morphological Feature	Common Associated Disorders	Clinical Interpretation
Sickle cells	Sickle cell disease	HbS polymerization
Target cells	Thalassemia, hemoglobinopathies	Excess membrane relative to cell volume
Spherocytes	Hereditary spherocytosis	Membrane surface loss
Elliptocytes	Hereditary elliptocytosis	Cytoskeletal abnormalities
Stomatocytes	Hereditary stomatocytosis	Membrane ion channel defects
Bite cells	G6PD deficiency	Splenic removal of Heinz bodies
Heinz bodies	G6PD deficiency, unstable hemoglobins	Oxidative denatured hemoglobin (requires supravital stain)
Basophilic stippling	Thalassemia, sideroblastic anemia	Abnormal erythropoiesis or RNA persistence
Nucleated RBCs	Severe hemolysis, thalassemia major, CDA	Intense marrow stimulation
Polychromasia	Hemolytic anemias	Increased reticulocyte production
Howell–Jolly bodies	Sickle cell disease, post-splenectomy	Hyposplenism or asplenia

**Table 17:** Major reticulocyte and hemolytic biomarkers in inherited anemias.

Biomarker	Physiological Role	Laboratory Finding	Diagnostic Significance
Reticulocyte Count	Measures bone marrow response	↑ Hemolysis; ↓ Ineffective erythropoiesis	Assesses erythropoietic activity
Corrected Reticulocyte Count (CRC)	Adjusts reticulocyte count for anemia severity	Low or high depending on marrow response	Improves interpretation
Reticulocyte Production Index (RPI)	Evaluates effective erythropoiesis	>2 regenerative; <2 inadequate marrow response	Differentiates hemolysis from marrow failure
Lactate Dehydrogenase (LDH)	Marker of cellular destruction	Elevated	Indicates ongoing hemolysis
Unconjugated Bilirubin	Product of heme degradation	Elevated	Supports chronic hemolysis
Haptoglobin	Binds free plasma hemoglobin	Decreased	Suggests intravascular hemolysis
Plasma Free Hemoglobin	Reflects circulating free Hb	Elevated	Indicates severe intravascular hemolysis
Hemoglobinuria/Hemosiderinuria	Renal excretion of hemoglobin/iron	Present	Supports intravascular hemolysis

**Table 18:** Interpretation of reticulocyte count in major inherited anemias.

Disease Category	Expected Reticulocyte Response	Interpretation
Hereditary Spherocytosis	Markedly increased	Active marrow compensation
Sickle Cell Disease	Increased	Chronic hemolysis
Pyruvate Kinase Deficiency	Increased	Chronic regenerative hemolysis
G6PD Deficiency	Increased during acute episodes	Oxidative hemolysis
Thalassemia Major	Variable; often lower than expected	Ineffective erythropoiesis predominates
Congenital Dyserythropoietic Anemia	Low or inappropriately normal	Bone marrow maturation defect
Diamond–Blackfan Anemia	Markedly reduced	Pure red cell aplasia

**Table 19:** Comparison of major hemoglobin analysis techniques.

Technique	Principle	Main Clinical Applications	Advantages	Limitations
Hemoglobin Electrophoresis	Separation based on electrical charge	Initial screening for hemoglobinopathies	Simple, inexpensive, widely available	Limited resolution; overlapping migration patterns
High-Performance Liquid Chromatography (HPLC)	Separation based on ionic interactions	Quantification of HbA_2_, HbF, HbS, HbC, HbE	Automated, highly reproducible, quantitative	Some variants have similar retention times
Capillary Electrophoresis (CE)	Separation in silica capillaries under electric field	Hemoglobin variant identification and quantification	High resolution, automation, rapid analysis	Requires specialized instrumentation
Isoelectric Focusing (IEF)	Separation according to isoelectric point	Newborn screening and variant detection	Excellent analytical resolution	Less commonly used in routine adult testing
Mass Spectrometry (MS)	Separation by molecular mass	Rare variant identification and confirmation	Extremely high analytical accuracy	High cost and limited availability

**Table 20:** Characteristic hemoglobin profiles in common inherited disorders.

Disorder	HbA	HbA_2_	HbF	Characteristic Finding
Normal Adult	95–98%	2–3.5%	<1%	Normal profile
β-Thalassemia Trait	Slightly reduced	↑ (>3.5%)	Mildly increased	Elevated HbA_2_
β-Thalassemia Major	Absent or markedly reduced	Variable	Markedly increased	Predominant HbF
Sickle Cell Trait (HbAS)	Present	Normal	Normal	HbA > HbS
Sickle Cell Disease/Anemia (HbSS)	Reduced/Absent	Normal	Increased	Predominant HbS
Hemoglobin C Disease	Reduced	Variable	Normal	Presence of HbC
Hemoglobin E Disease	Reduced	Mildly increased	Variable	Presence of HbE
Hereditary Persistence of Fetal Hemoglobin (HPFH)	Present	Normal	Markedly increased	Persistent HbF

**Table 21:** Specialized laboratory assays used in inherited anemia diagnosis.

Laboratory Assay	Primary Target	Major Clinical Applications	Advantages	Limitations
Osmotic Fragility Test (OFT)	Membrane stability	Hereditary spherocytosis screening	Simple, inexpensive	Limited sensitivity and specificity
EMA Binding Test	Band 3 membrane protein	Hereditary spherocytosis	High sensitivity and specificity	Requires flow cytometer
Osmotic Gradient Ektacytometry	Erythrocyte deformability	Membrane disorders	Excellent diagnostic accuracy	Limited availability
Enzyme Activity Assays	Intracellular enzyme function	G6PD, PK deficiency, other enzymopathies	Gold standard for enzyme disorders	Influenced by transfusion and reticulocytosis
Flow Cytometry	Membrane proteins and cellular markers	Membrane disorders, reticulocytes	Rapid, objective, multiparametric	Specialized equipment required
Bone Marrow Examination	Erythroid morphology	CDA, DBA, Fanconi anemia, sideroblastic anemia	Direct assessment of erythropoiesis	Invasive procedure
Iron Studies	Iron metabolism	Thalassemia, iron overload, differential diagnosis	Widely available	Ferritin affected by inflammation

**Table 22:** Comparison of major molecular diagnostic techniques.

Technique	Detects	Major Applications	Advantages	Limitations
PCR	Known mutations	Targeted mutation screening	Rapid, inexpensive	Limited to known variants
Sanger Sequencing	SNVs, small indels	Variant confirmation	High accuracy	Low throughput
MLPA	Copy number variations	α-thalassemia, gene deletions	Detects deletions/duplications	Does not detect SNVs
NGS Gene Panels	Multiple disease genes	Routine inherited anemia diagnosis	High diagnostic yield	Requires bioinformatics
WES	Coding regions	Undiagnosed inherited anemia	Broad genomic coverage	Misses many non-coding variants
WGS	Entire genome	Complex genetic disorders	Most comprehensive	Expensive; large data burden
Third generation sequencing	Long structural variants	Complex rearrangements, repetitive regions	Long-read accuracy	Limited availability

**Table 23:** Major components of supportive management in inherited anemias.

Supportive Intervention	Primary Purpose	Common Clinical Indications
Red Blood Cell Transfusion	Correct severe anemia and improve oxygen delivery	β-thalassemia major, DBA, CDA, severe PK deficiency
Folic Acid Supplementation	Support accelerated erythropoiesis	Chronic hemolytic anemias
Vaccination	Prevent overwhelming infections	Asplenia, splenectomy, sickle cell disease
Antibiotic Prophylaxis	Reduce bacterial infections	Children with sickle cell disease, post-splenectomy patients
Clinical Monitoring	Detect organ complications	All inherited anemias
MRI T2*	Assess cardiac and hepatic iron overload	Transfusion-dependent patients
Psychological and Nutritional Support	Improve quality of life	Chronic inherited anemia
Patient Education	Enhance adherence and complication prevention	All inherited anemias

**Table 24:** Clinical indications for blood transfusion in major inherited anemias.

Disease	Primary Indication	Preferred Strategy
β-Thalassemia Major	Lifelong chronic anemia	Regular simple transfusion
Sickle Cell Disease	Stroke prevention, acute chest syndrome, severe anemia	Exchange or simple transfusion
Diamond–Blackfan Anemia	Severe congenital anemia	Regular simple transfusion
Congenital Dyserythropoietic Anemia	Severe ineffective erythropoiesis	Individualized transfusion
Pyruvate Kinase Deficiency	Severe chronic hemolysis	Symptom-based transfusion

**Table 25:** Comparison of currently available iron chelators.

Chelator	Route	Major Advantages	Main Limitations
Deferoxamine (DFO)	Subcutaneous/IV	Highly effective	Prolonged infusion; poor adherence
Deferiprone (DFP)	Oral	Excellent cardiac iron removal	Agranulocytosis risk
Deferasirox (DFX)	Oral	Once-daily dosing; improved adherence	Renal and hepatic monitoring required

**Table 26:** Major disease-modifying drugs in inherited anemias.

Drug	Disease	Mechanism of Action	Principal Clinical Benefit
Hydroxyurea	Sickle cell disease	Increases fetal hemoglobin (HbF)	Reduces vaso-occlusive crises and mortality
Luspatercept	β-Thalassemia	Promotes erythroid maturation	Reduces transfusion burden
Mitapivat	Pyruvate kinase deficiency	Activates pyruvate kinase enzyme	Improves hemoglobin and decreases hemolysis
Voxelotor	Sickle cell disease	Increases hemoglobin oxygen affinity, reducing HbS polymerization	Withdrawn from market in 2024 because of safety concerns; not a current treatment option
Crizanlizumab	Sickle cell disease	Anti-P-selectin monoclonal antibody	P-selectin inhibitor developed to reduce vaso-occlusive crises in sickle cell disease. In the European Union, Adakveo had been conditionally authorized for prevention of recurrent vaso-occlusive crises in patients with sickle cell disease aged ≥ 16 years, either as add-on therapy to hydroxyurea/hydroxycarbamide or as monotherapy where such therapy was inappropriate or inadequate. The European Commission revoked the EU marketing authorization on 3 August 2023 after the confirmatory STAND study failed to confirm clinical benefit. In STAND, the adjusted annualized rate of vaso-occlusive crises leading to a healthcare visit was 2.49 (95% CI 1.90–3.26) with crizanlizumab 5 mg/kg versus 2.30 (1.75–3.01) with placebo (rate ratio 1.08, 95% CI 0.76–1.55; adjusted *p* > 0.999).

**Table 27:** Clinical applications of HSCT in inherited anemias.

Disease	Indication	Expected Outcome
β-Thalassemia Major	Transfusion dependence	Potentially curative in appropriately selected patients
Sickle Cell Disease	Severe vaso-occlusive complications, stroke	Potentially curative in appropriately selected patients
Diamond–Blackfan Anemia	Steroid-resistant transfusion dependence	Potentially curative in appropriately selected patients
Fanconi Anemia	Progressive bone marrow failure	Potentially curative in appropriately selected patients
Congenital Dyserythropoietic Anemia	Severe transfusion-dependent disease	Potentially curative

**Table 28:** Approved and emerging gene therapies.

Therapy	Disease	Therapeutic Strategy	Clinical Outcome
Betibeglogene autotemcel (Zynteglo^®^)	β-Thalassemia	Lentiviral β-globin gene addition	Transfusion independence
Lovotibeglogene autotemcel (Lyfgenia^®^)	Sickle Cell Disease	Modified β-globin gene addition	FDA-authorized for defined SCD population; boxed warning for hematologic malignancy
Experimental Lentiviral Therapies	Rare inherited anemias	Gene replacement	Ongoing clinical trials
Exagamglogene autotemcel (Casgevy^®^)	Sickle cell disease; transfusion-dependent β-thalassemia	Ex vivo CRISPR/Cas9 editing of the BCL11A erythroid enhancer	Authorized for defined populations; durability and late safety require continued follow-up

**Table 29:** Emerging genome editing technologies.

Technology	Principle	Major Advantages	Current Status
CRISPR-Cas9	Double-strand DNA editing	Clinically validated ex vivo editing for selected hemoglobinopathies	Authorized for defined indications via exagamglogene autotemcel
Base Editing	Single nucleotide conversion	Potential single-base correction without double-strand breaks	Investigational
Prime Editing	Precise DNA replacement	Potentially precise sequence replacement	Preclinical/early investigational
Epigenome Editing	Gene regulation without DNA sequence change	Modulates gene expression without changing DNA sequence	Preclinical/early research

**Table 30:** Summary of the literature selection process.

Literature Selection Stage	Number of Papers
Records identified	5700
Duplicates removed	1278
Records screened by title	4422
Records excluded by title	3498
Abstracts assessed	924
Abstracts excluded	600
Full-text articles assessed	324
Full-text/final relevance articles excluded	255
Studies included in narrative synthesis	69

**Table 31:** Distribution of publications included in the final evidence synthesis by evidence domain (n = 69).

Evidence Domain	Number of Publications (n)	Percentage (%)
Clinical Medicine	11	15.9
Laboratory Hematology	21	30.4
Molecular Genetics	16	23.2
Cross-cutting/Multidomain Evidence	21	30.4
Total	69	100

**Table 32:** Principal evidence publications supporting the key findings.

Ref.	Evidence Contribution Identifiable from your Manuscript	Classification
[2]	Molecular diagnosis of hereditary hemolytic anemias	Molecular/diagnostic evidence
[3]	NGS in diagnosis of rare inherited anemias	Molecular/diagnostic evidence
[5]	Genetics/genomics approaches for hereditary anemia; overlapping phenotypes	Cross-cutting/molecular evidence
[6]	NGS/focused genetic testing in congenital anemias; differentiation of overlapping disorders	Diagnostic/molecular evidence
[8]	Molecular findings/causal genes in congenital dyserythropoietic anemia	Molecular evidence
[9]	Thalassemia clinical and diagnostic characteristics	Clinical/diagnostic evidence
[10]	Sickle cell disease diagnosis and molecular testing	Clinical/molecular evidence
[11]	Sickle cell diagnostic developments and laboratory investigation	Laboratory/diagnostic evidence
[12]	Congenital hemolytic anemia; HS, EMA testing, G6PD and PK deficiency	Clinical/laboratory evidence
[13]	Clinical/laboratory evidence for congenital dyserythropoietic anemia	Clinical/laboratory evidence
[14]	NGS for thalassemia screening/diagnosis	Molecular/diagnostic evidence
[15]	Hemoglobin electrophoresis in diagnosis	Laboratory evidence
[16,17,18]	NGS/TGS and structural-variant detection in inherited anemia	Molecular evidence

## Data Availability

No new data were created or analyzed in this study. Data sharing is not applicable to this article.
